# Tuning the Affinity of Chimeric Antigen Receptors Enhances the Function of Human Engineered Regulatory T Cells

**DOI:** 10.1002/eji.70226

**Published:** 2026-06-17

**Authors:** Ada Sera Kurt, Marwa Elgosbi, Elisavet Kodela, Emmanuelle Landmann, Paula Ruiz, Yoh Zen, Jorge Torres‐Yaguana, Alexander Puttick, Quisha Bustamante, Minahil Sharjeel, Hannan Murshid, Marc Martinez‐Llordella, Alberto Sanchez‐Fueyo

**Affiliations:** ^1^ Roger Williams Institute of Liver Studies School of Immunology & Microbial Sciences (SIMS) King's College London University and King's College Hospital London UK; ^2^ Institute of Liver Studies King's College Hospital London UK; ^3^ Quell Therapeutics London UK

## Abstract

Chimeric antigen receptors (CAR) incorporating single‐chain variable fragments (scFv) exhibit much higher affinity for their cognate antigens than native T cell receptors (TCR). Reducing the affinity of the CAR has been shown to improve the function of effector CAR‐T cells by limiting their activation‐induced exhaustion while preserving their capacity for serial killing of antigen‐rich tumor cells. CAR technologies are increasingly being applied to regulatory T cell (Treg)–based immunotherapies as well. However, the impact of CAR affinity on Treg biology and function remains poorly understood. To address this question, we transduced purified human Tregs with second‐generation CAR constructs bearing scFvs with varying affinities toward HLA‐A2 and compared their properties both in vitro and in vivo. High‐affinity (HA) CAR‐Tregs displayed higher avidity and more pronounced CAR downregulation upon antigen engagement. In contrast, low‐affinity (LA) CAR‐Tregs exhibited enhanced antigen‐specific activation and superior suppressive capacity. These differences were confirmed using human and mouse precision‐cut liver slices and a xenogeneic graft‐versus‐host disease (GVHD) murine model. LA CAR‐Tregs exhibited greater accumulation/persistence, delayed GVHD onset, and improved survival than HA CAR‐Tregs. Our findings, indicating that CAR affinity strongly influences CAR‐Treg function, provide important considerations for the optimization of engineered Treg therapies and the benchmarking of existing cell products.

## Graphical abstract

Affinity tuning of anti‐HLA‐A2 CARs reveals that lower‐affinity CAR‐Tregs exhibit reduced avidity and enhanced serial engagement, leading to increased activation and trogocytosis. These properties promote improved tissue infiltration, persistence, and survival in vivo, demonstrating that optimizing receptor–antigen dynamics enhances the therapeutic efficacy of engineered regulatory T cells.

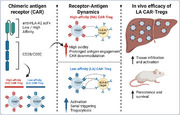

## 1 Introduction

CD4^+^CD25^+^Foxp3^+^ regulatory T cells (Tregs), which constitute 5%–10% of the circulating CD4^+^ T cell compartment [1], are essential to maintain immune homeostasis and prevent fatal autoimmunity. Tregs exert powerful anti‐inflammatory effects via a range of nonredundant mechanisms, such as production of immunosuppressive cytokines, IL‐2 sequestering, and the removal of peptide‐MHC complexes and costimulatory molecules from antigen‐presenting cells (APCs) via trogocytosis [2, 3, 4, 5]. Given the feasibility of generating a large number of Tregs in the laboratory, Treg therapies hold promise as novel strategies to ameliorate immunopathology and re‐establish immune tolerance in autoimmune diseases and transplantation. Autologous polyclonal (PC) Tregs have already been proven to be safe in patients with graft‐versus‐host disease (GvHD) [6, 7, 8], type 1 diabetes [9], and organ transplantation [10], and have been shown to exert beneficial clinical effects in GvHD [11].

The activation of Tregs requires cognate antigen engagement through their T cell receptor (TCR), following which their immunosuppressive effects affect all cells in their vicinity (bystander suppression). Accordingly, Tregs with specificity for antigens expressed in a particular tissue compartment (e.g., the transplanted graft) preferentially traffic to and persist in this site [12, 13, 14, 15], which results in more powerful immunoregulatory effects than when PC‐Tregs with no predefined antigen specificity are employed. This is the reason why synthetic biology approaches capable of artificially conferring antigen‐specificity through the expression of chimeric antigen receptors (CAR) have gained significant momentum in the field of Treg immunotherapy [16]. CARs, which are usually designed to enable MHC‐independent recognition of surface antigens, consist of an extracellular antigen‐binding domain, hinge, transmembrane domain, and intracellular signaling domains. Each of these components can influence the functional properties of the CAR. This has been extensively studied for CAR‐T but much less for CAR‐Tregs.

The CAR hinge domain connects the antigen‐binding domain to the transmembrane domain and provides flexibility, allowing the scFv to access specific epitopes. Shorter hinge regions may enhance cell activation by facilitating antigen engagement and promoting stronger intracellular signaling cascades. Conversely, longer hinges may increase tonic signaling and off‐target effects through Fc receptor interactions [17, 18]. The transmembrane domain, which anchors the CAR to the T cell membrane and is involved in synapse formation, can mediate interactions with endogenous signaling molecules and has an impact on CAR expression level and stability [19]. The intracellular signaling domains typically include CD3ζ and one or more costimulatory domains. Effector T cells expressing CARs containing 41BB and/or TNRF2 exhibit better in vivo persistence than those incorporating CD28 [20, 21]. In contrast, CD28‐based CARs are superior to other CARs in terms of CAR‐Treg lineage stability, FOXP3 expression, and suppressive capacity [22]. The most commonly used antigen‐binding domain is a single‐chain variable fragment (scFv) formed by a heavy (VH) and a light (VL) variable antibody fragment [23]. CARs containing an scFv exhibit an affinity for their target antigens that is several orders of magnitude higher than that of a TCR. While TCRs have dissociation constants (K_D_) in the micromolar range, scFv‐containing CARs typically exhibit affinities <20 nanomolar [24]. Altering the antigen‐binding affinity of CAR‐expressing effector T cells can profoundly affect both their specificity and potency. In particular, lowering the CAR affinity has been shown to increase CAR‐T antitumor selectivity by reducing off‐tumor and/or off‐target activation [20] and to enhance antitumor cytotoxicity [25]. This was confirmed in a clinical trial in pediatric acute lymphoblastic leukemia (ALL) in which the use of an anti‐CD19 CAR‐T cell product exhibiting lower affinity than FMC63 (the binder employed in most clinically used CAR‐T therapies) increased expansion and persistence, reduced exhaustion, and resulted in improved clinical outcomes [26]. However, several studies demonstrate that the functional consequences of CAR affinity are indeed highly dependent on target antigen density. In settings of low antigen expression, high‐affinity scFvs confer superior CAR‐T effector function compared with low‐affinity constructs, as shown in ROR1‐expressing epithelial cancers and FRβ‐positive acute myeloid leukemia models [27, 28].

In contrast to CAR‐T cells, the extent to which CAR affinity influences CAR‐Treg function remains to be thoroughly clarified. Two ongoing clinical trials are currently evaluating the safety and efficacy of anti‐HLA‐A2 CAR‐Tregs in liver and kidney transplant recipients who are mismatched for HLA‐A2 (LIBERATE trial; NCT05234190 and STEADFAST trial; NCT04817774, respectively). HLA‐A2 was selected as a target given that 25% of transplant recipients are mismatched for HLA‐A2. Furthermore, preclinical studies have demonstrated that HLA‐A2‐specific CAR‐Tregs are stable and effective in humanized mouse models of GvHD and skin transplantation [29, 30, 31]. In the current study, we chose to employ the same target to investigate the effects of CAR affinity tuning on the functional properties of CAR‐Tregs by modifying the anti‐HLA‐A2 scFv domain. We generated three anti‐HLA‐A2 CAR‐Treg products, expressing a scFv antigen‐binding domain with low, moderate, and high‐affinity, respectively, and evaluated their functional properties in vitro and in vivo. Our results suggest that lowering the affinity of scFvs toward their cognate antigen enhances the function of CAR‐Tregs and their capacity to infiltrate human tissues. These findings provide valuable insight into optimizing the design of future engineered Treg therapies for clinical use.

## 2 Results

### 2.1 Generation of Functional CAR‐Tregs With Varying Affinities Toward HLA‐A2

**FIGURE 1:**
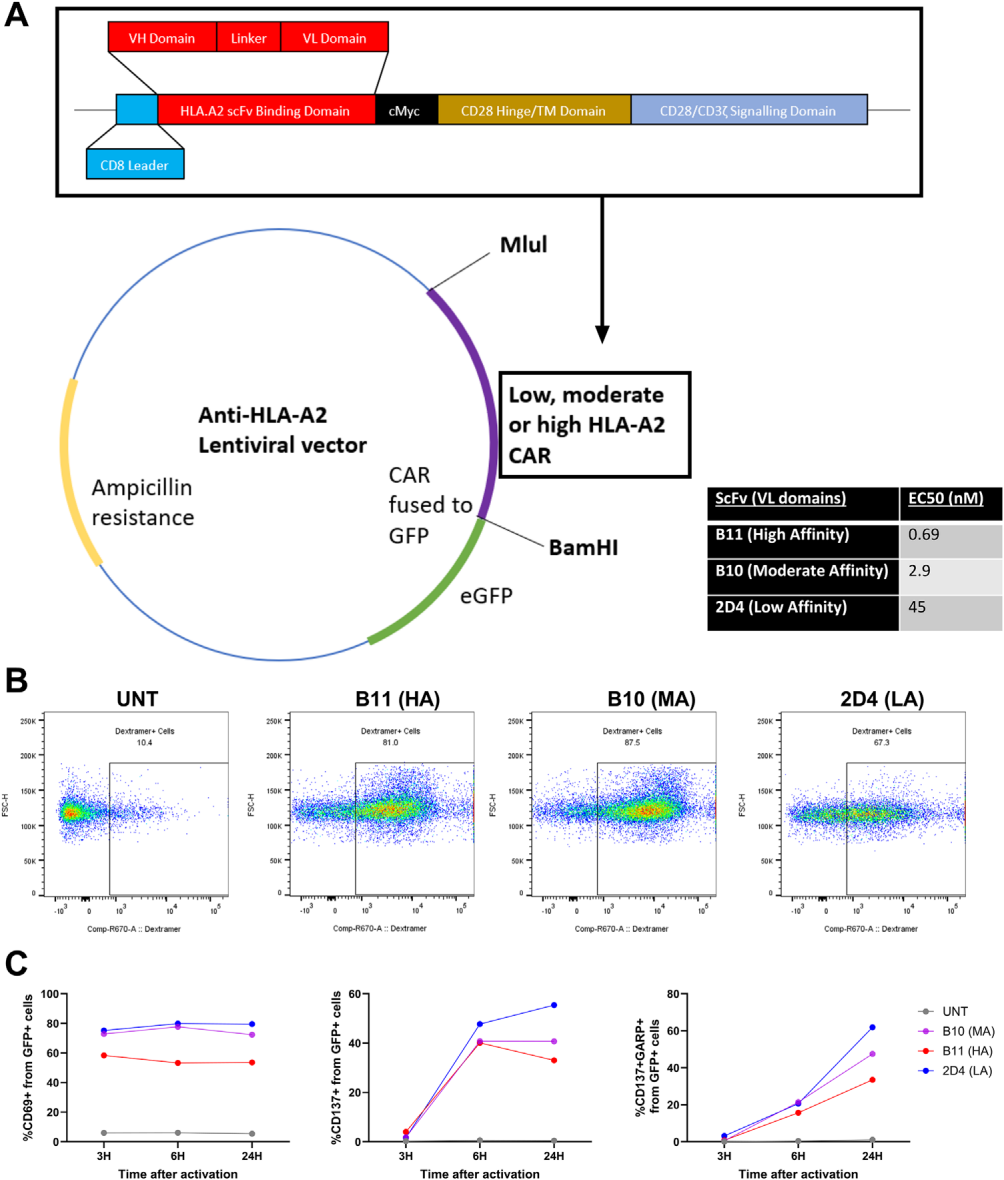
Design, surface expression, and function of the three anti‐HLA‐A2 CARs. (A) Schematic and plasmid map of the second‐generation HLA‐A2 CAR constructs (low, moderate, and high affinity) used in the study. Components include CD8 leader, HLA‐A2 scFv (VH and VL domains), C‐Myc tag, CD28 extracellular and transmembrane domains, and CD3ζ signaling domain. This was fused to the eGFP reporter sequence (not shown). EC50 values for the VL scFv antigen‐binding domains were determined by titration using HLA‐A2‐positive platelets as described by Watkins et al. [51]. (B) Representative dot plots from three independent experiments, 3 days posttransduction, displaying the proportion of dextramer^+^ Tregs 3 days after transduction with the B11, B10, and 2D4 constructs. (C) Time‐lapse expression of activation markers (CD69, CD137, and CD137^+^GARP^+^) on untransduced Tregs and different CAR‐Treg preparations following culture with HLA‐A2‐expressing EBV‐transformed B cells. Each data point represents the mean of three replicates from a single donor, measured at the indicated time points. UNT; untransduced, 2D4 (LA); low‐affinity, B10 (MA); moderate‐affinity, and B11 (HA); high‐affinity.

Second‐generation HLA‐A2 CARs expressing low, moderate, or high‐affinity scFv antigen‐binding domains were designed and cloned into lentiviral plasmids containing an eGFP gene fused to the CD3ζ signaling domain. CAR‐Tregs were generated via lentiviral transduction of sorted HLA‐A2^−^ CD4^+^CD25^+^ CD127^lo^ Tregs (Figure 1A; Figure S1). We used a fluorochrome‐conjugated HLA‐A2 dextramer to assess the surface expression of the CAR, which was found to be similar in all 3 cell types. (Figure 1B; Table S1). We next employed an activation‐induced marker assay that included CD69, CD137, and GARP to track the activation of the cells over time in response to an HLA‐A2^+^ cell line. While all three types of CAR‐Tregs rapidly upregulated activation markers following stimulation with HLA‐A2, we observed a clear gradation in the level of response, with 2D4 exhibiting the highest expression of activation markers, B11 the lowest, and B10 intermediate levels (Figure 1C; Table S2). Based on these results, we decided to focus for all subsequent experiments on the comparison between 2D4 (low‐affinity or LA) and B11 (high‐affinity or HA).

### 2.2 ScFv Affinity Does Not Influence the Phenotype of CAR‐Tregs Upon Stimulation

**FIGURE 2:**
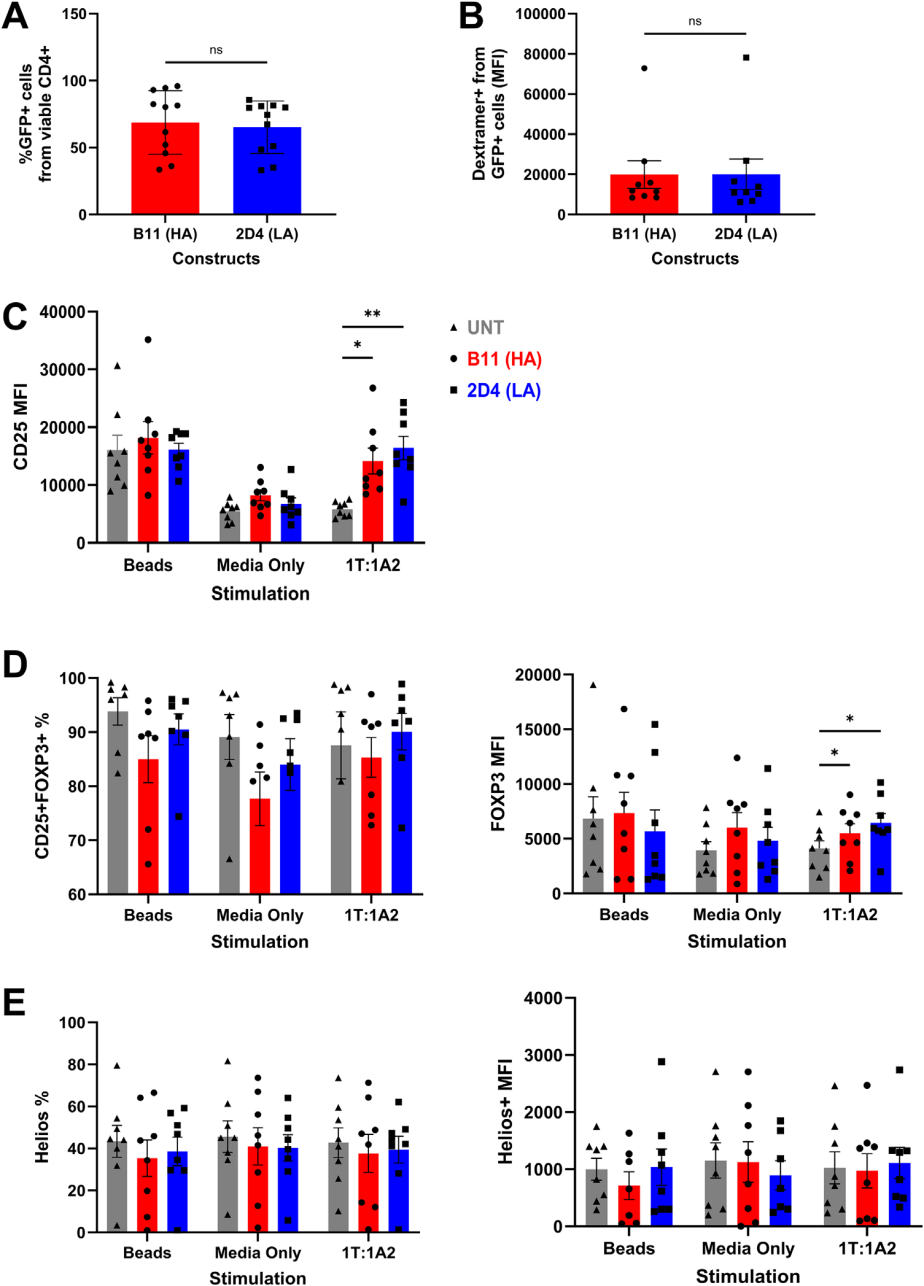
CAR affinity does not impact the phenotypic characteristics of CAR‐Tregs upon stimulation. (A) Percentage of GFP+ cells from viable CD4+ T cells. (B) Mean fluorescent intensity (MFI) of dextramer‐positive cells among GFP^+^ cells (*n* = 11). (C–E) Phenotypic characteristics of Treg products (*n* = 8) after 5 days of culture with either media (no activation), CD3/CD28 beads (TCR activation), or an HLA‐A2‐expressing cell line (CAR activation). Plots display CD25 MFI (C), proportion of CD25^+^FOXP3^+^ cells and their FOXP3 MFI (D), and proportion of Helios^+^ cells and their Helios MFI (E). All proportions correspond to Tregs gated on viable CD4^+^ T cells (*n* = 8); values depict mean± SEM. Statistical analyses were conducted using a paired *t*‐test (A, B) and a repeated measures two‐way ANOVA with a Tukey's multiple comparison test (C, E). *p < 0.05, **p < 0.01. UNT: untransduced Tregs; 2D4 (LA): low‐affinity CAR‐Tregs; B11 (HA): high‐affinity CAR‐Tregs.

First, we aimed to determine whether LA and HA CAR‐Tregs exhibit consistent transduction efficacies and similar proportions of HLA‐A2‐specific cells. This was important to ensure that any functional differences observed would be solely due to the varying affinities of the constructs, rather than differences in transduction efficacy or proportion of antigen‐specific cells generated following transduction. The two constructs did not differ in the overall proportion of GFP^+^ transduced cells (68.79% for B11 HA and 65.2% for 2D4 LA CAR‐Tregs; Figure 2A). Additionally, the magnitude of CAR expression, as assessed by the levels of dextramer^+^ staining, was also similar (pooled MFI of 19941 for HA and 20064 for LA; Figure 2B; Figure S2). We next investigated how the choice of CAR would influence the phenotype and lineage stability of CAR‐Tregs following cognate antigen exposure. For this purpose, we co‐cultured either untransduced, LA, or HA CAR‐Tregs with HLA‐A2‐expressing cell lines and characterized their phenotype after 5 days of culture. As compared with untransduced Tregs, both LA and HA CAR‐Tregs significantly increased CD25^+^ (Figure 2C) and FOXP3^+^ expression (Figure 2D). Furthermore, both CAR‐Tregs maintained stable Helios expression and retained their regulatory phenotype following stimulation through either their CAR or TCR (Figure 2D,E).

### 2.3 Low‐Affinity CAR‐Tregs Exhibit Enhanced Antigen‐Specific Activation and Suppression

**FIGURE 3:**
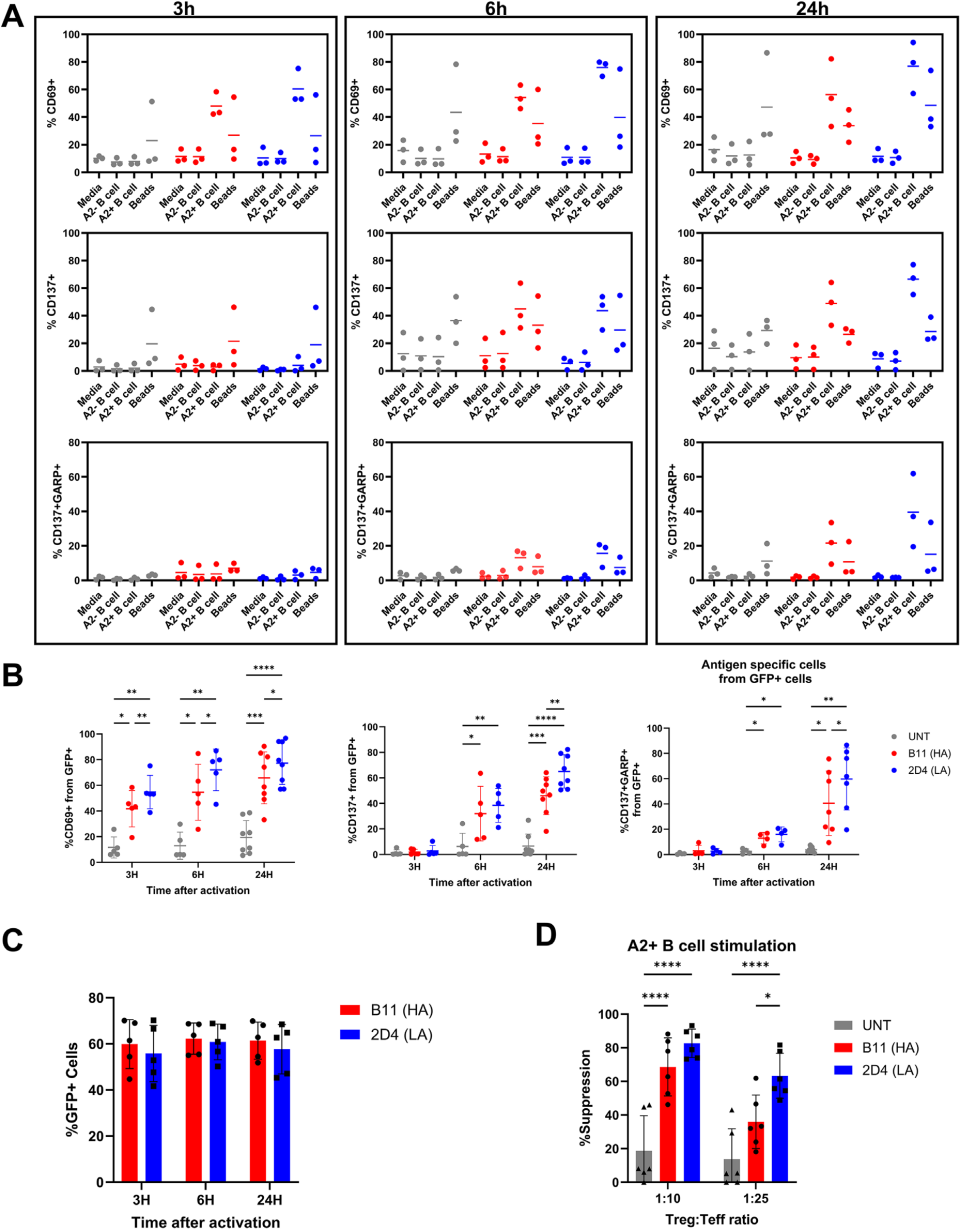
Low‐affinity anti‐HLA‐A2 CAR‐Tregs exhibit a higher proportion of cells activated following cognate antigen recognition and exert the most powerful suppressive effects. (A) Percentage of CD4^+^ GFP^+^ cells exhibiting CD69, CD137, and CD137/GARP activation markers after 3, 6, and 24 h of culture with either media, HLA‐A2‐expressing B cells, or CD3/CD28 beads, as assessed by flow cytometry. Data correspond to 3 independent experiments; horizontal lines depict median values. (B) Scatter plot derived from flow cytometry data depicting the Time‐lapse expression of activation markers (CD69, CD137, CD137 and GARP) on untransduced (UNT), B11 CAR‐Tregs (high‐affinity, in red), and 2D4 CAR‐Tregs (Low‐affinity, in blue) CAR‐T, after culture with HLA‐A2‐expressing EBV‐transformed B cells (*n* = 7). (C) Proportion of GFP^+^ cells from viable CD4^+^ T cells at different times postactivation (*n* = 5). (D) Suppressive capacity of different Treg preparations at variable Treg: T‐eff ratios, evaluated at day 5 (*n* = 6). Values in (B, D) correspond to mean ± SD. Analyses were conducted using mixed‐effects models (B) or two‐way ANOVA with Tukey's multiple comparison test correction (C, D). *p < 0.05, **p < 0.01, ***p < 0.001, ****p < 0.0001. UNT: untransduced Tregs; 2D4 (LA): low‐affinity CAR‐Tregs; B11 (HA): high‐affinity CAR‐Tregs.

We then evaluated the kinetics of activation of LA and HA CAR‐Tregs in response to either TCR or CAR stimulation. These results confirmed that neither the LA nor the HA CAR constructs provided a significant degree of tonic signaling. Furthermore, when cultured with anti‐CD3/CD28 beads, both CAR‐Tregs exhibited a similar activation profile to untransduced Tregs, indicating that the CAR transduction did not modify their functionality (Figure 3A). In response to HLA‐A2^+^ EBV‐B cell stimulation, both LA and HA CAR‐Tregs upregulated CD69 expression as early as 3 h after initiating the culture, reaching a peak at 24 h. However, LA CAR‐Tregs consistently exhibited a higher activation level than HA CAR‐Tregs (*p* < 0.01 at 3 h; *p* < 0.05 at 6 and 24 h). The markers of Treg late‐stage activation, CD137 and GARP, were noticeable 6 h poststimulation, and by 24 h their expression was significantly higher in LA CAR‐Tregs than in HA CAR‐Tregs (*p* < 0.05 at 6 h and *p* < 0.01 at 24 h) (Figure 3B; Figures S3 and S4). Of note, the percentage of transduced cells did not differ significantly between constructs at the different time points analyzed after activation (Figure 3C). In keeping with the results of the activation assay, LA CAR‐Tregs exhibited a significantly greater suppressive capacity than B11 HA CAR‐Tregs in the presence of HLA‐A2 antigen stimulation, with nonantigen‐specific unstimulated Tregs displaying almost negligible suppressive effects (Figure 3D; Figure S5 and Table S3).

### 2.4 Anti‐HLA‐A2 CAR Affinity Regulates the Functionality of Tregs Infiltrating Human and Mouse Liver Tissues

**FIGURE 4:**
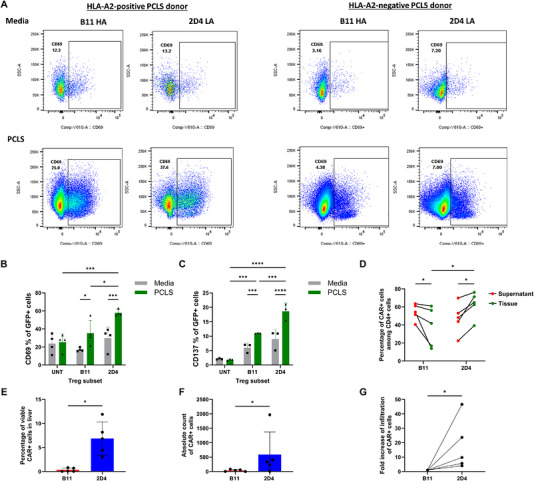
Activation and tissue infiltration of low‐ and high‐affinity CAR‐Tregs in a precision‐cut liver slice model (PCLS). (A) Representative flow cytometry dot plots showing CD69 expression among GFP^+^ cells from HA and LA CAR‐Treg preparations following 18 h of culture with HLA‐A2^+^ or HLA‐A2^−^ PCLS. (B, C) Proportion of CD69^+^ and CD137^+^ among GFP^+^ untransduced and CAR‐Tregs products (*n* = 4). (D) Proportion of GFP^+^ cells among the total number of CD4^+^ T cells obtained from the supernatant or the homogenized liver tissue following 18 h of culture (*n* = 5). (E) Proportion of CAR^+^ cells among leukocytes obtained from the homogenized liver tissue following 18 h of culture. (F, G) Absolute number and fold‐increase of CAR^+^ cells obtained from the homogenized liver. Each dot corresponds to an independent Treg and PCLS donor. Boxes correspond to mean values, and whiskers show ± SD. Statistical analyses comprised repeated‐measure two‐way ANOVA with Tukey's multiple comparison test (B, D), paired *t*‐test (E), and Wilcoxon matched‐pairs signed rank test (F, G). *p < 0.05, **p < 0.01, ***p < 0.001, ****p < 0.0001. UNT: untransduced Tregs; 2D4 (LA): low‐affinity CAR‐Tregs; B11 (HA): high‐affinity CAR‐Tregs.

To explore if CAR affinity also influences Treg function when CAR‐Tregs encounter their target antigen at physiological densities within tissues, we conducted additional experiments utilizing precision‐cut liver slices (PCLS) obtained from either HLA‐A2^+^ human livers or livers from HLA‐A2 transgenic mice. Anti‐HLA‐A2 CAR‐Tregs upregulated CD69 expression after 18 h of culture with HLA‐A2^+^, but not HLA‐A2^−^, human PCLS (Figure 4A; Figure S6 and Table S4). In agreement with the in vitro experiments using cell lines, LA CAR‐Tregs exhibited a significantly higher level of activation than HA CAR‐Tregs (mean CD69^+^ among GFP^+^ cells 57.8% versus 35.4%, respectively; *p* < 0.05). A similar trend was observed for CD137 expression (Figure 4B,C). We then investigated if the activation conferred by the CAR resulted in different degrees of tissue infiltration and/or persistence. This was assessed by culturing HA and LA CAR Tregs with PCLS from HLA‐A2 transgenic mice. Following 18 h culture, the proportion of GFP+ cells among the overall population of LA CAR Tregs isolated from the PCLS significantly increased as compared with the cells collected from the supernatant, indicating an increased infiltration and/or selective retention of CAR‐expressing Tregs. The reverse was observed for HA CAR‐Tregs, in which only a small percentage of CAR‐expressing Tregs was observed when analyzing the tissue‐infiltrating Tregs (Figure 4D,E). The results were similar when expressed as an absolute number of tissue‐infiltrating CAR‐Tregs (Figure 4F). The overall differences between the LA and HA CARs were even more clearly illustrated by conducting a paired analysis comparing LA and HA CAR‐Tregs manufactured from matched donors (Figure 4G). A small set of experiments conducted using human PCLS revealed similar results (Figure S7).

### 2.5 Infusion of LA CAR‐Tregs Prolongs Recipient Survival in a Xenogeneic GvHD Humanized Mouse Model

**FIGURE 5:**
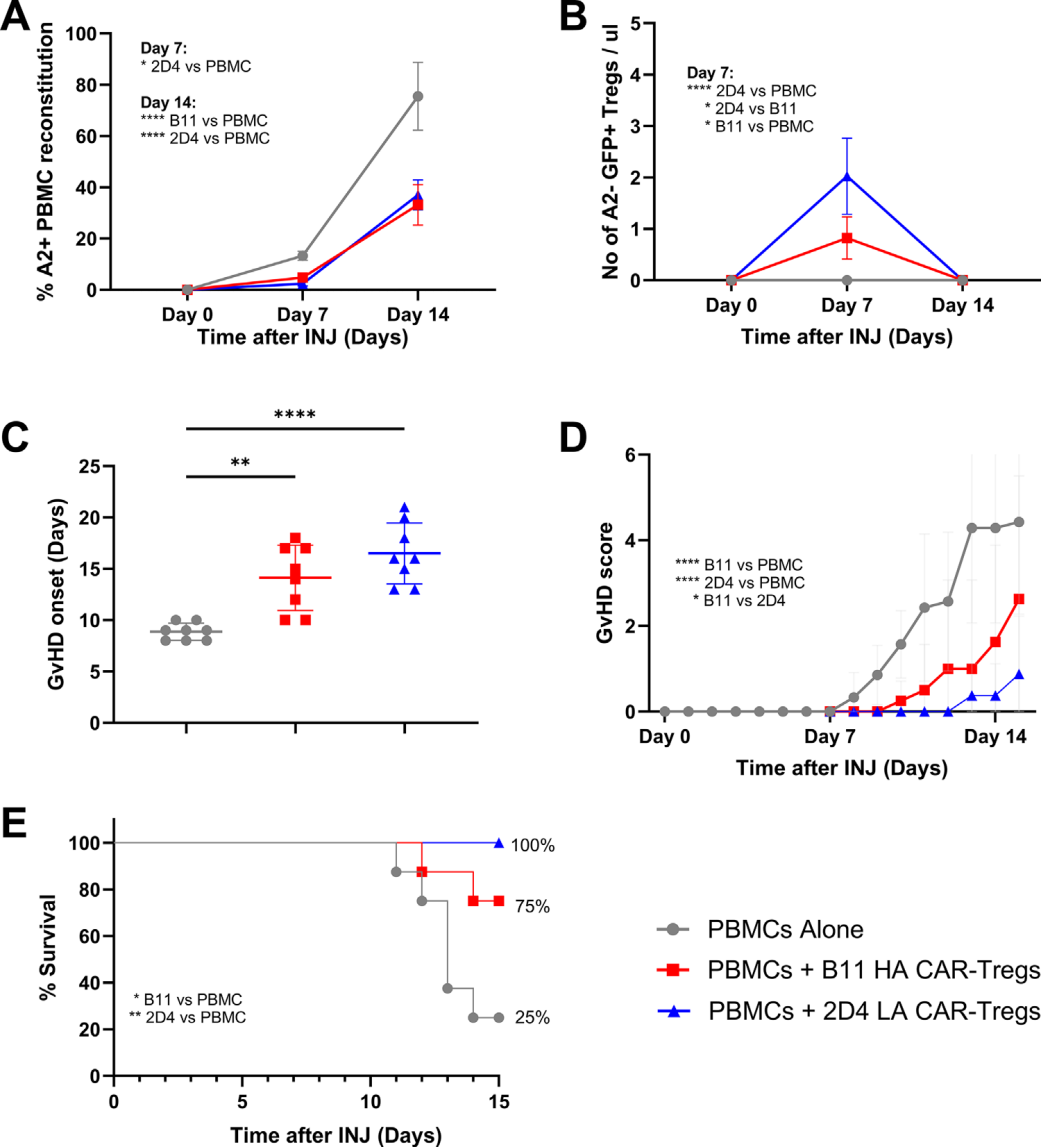
Effects of CAR affinity on the function of CAR‐Tregs in an in vivo xeno‐GvHD model. (A) Proportion of HLA‐A2^+^ human PBMCs among circulating CD45^+^ cells at different time points after cell infusion. (B) Absolute number of HLA‐A2^−^ GFP^+^ Tregs per µL of blood at different time points after cell infusion. (C) Day of GvHD onset. (D) Daily GvHD score until day 15 after cell infusion. (E) Kaplan–Meier analysis of 15‐day recipient survival (*n* = 8, three independent Treg donors). Values shown in (A–C) correspond to mean ± SD. Statistical analyses were performed using one‐way ANOVA (C) or two‐way ANOVA with Tukey's multiple comparison test (A, B, D). *p < 0.05, **p < 0.01, ****p < 0.0001.

We sought to elucidate the in vivo properties of LA and HA CAR‐Tregs. We employed a xenogeneic GvHD model in which immunodeficient NSG mice were reconstituted with human HLA‐A2^+^ PBMCs and received no Tregs, LA CAR‐Tregs, or HA CAR‐Tregs. Following infusion, LA CAR‐Tregs persisted for longer and suppressed the reconstitution of HLA‐A2+ PBMCs to a much greater extent than HA CAR‐Tregs (Figure 5A,B). We monitored their effects on the onset and progression of GvHD during the first 14 days after infusion (neither of the two CAR‐Treg products was detectable anymore following 14 days). As compared with HA CAR‐Treg‐treated mice, those receiving LA CAR‐Tregs exhibited delayed GvHD onset and progression (Figure 5C,D), as well as prolonged survival (Figure 5E).

### 2.6 CAR Affinity Correlates With Overall CAR‐Treg Avidity Across Different Cognate Antigen Density Levels

**FIGURE 6:**
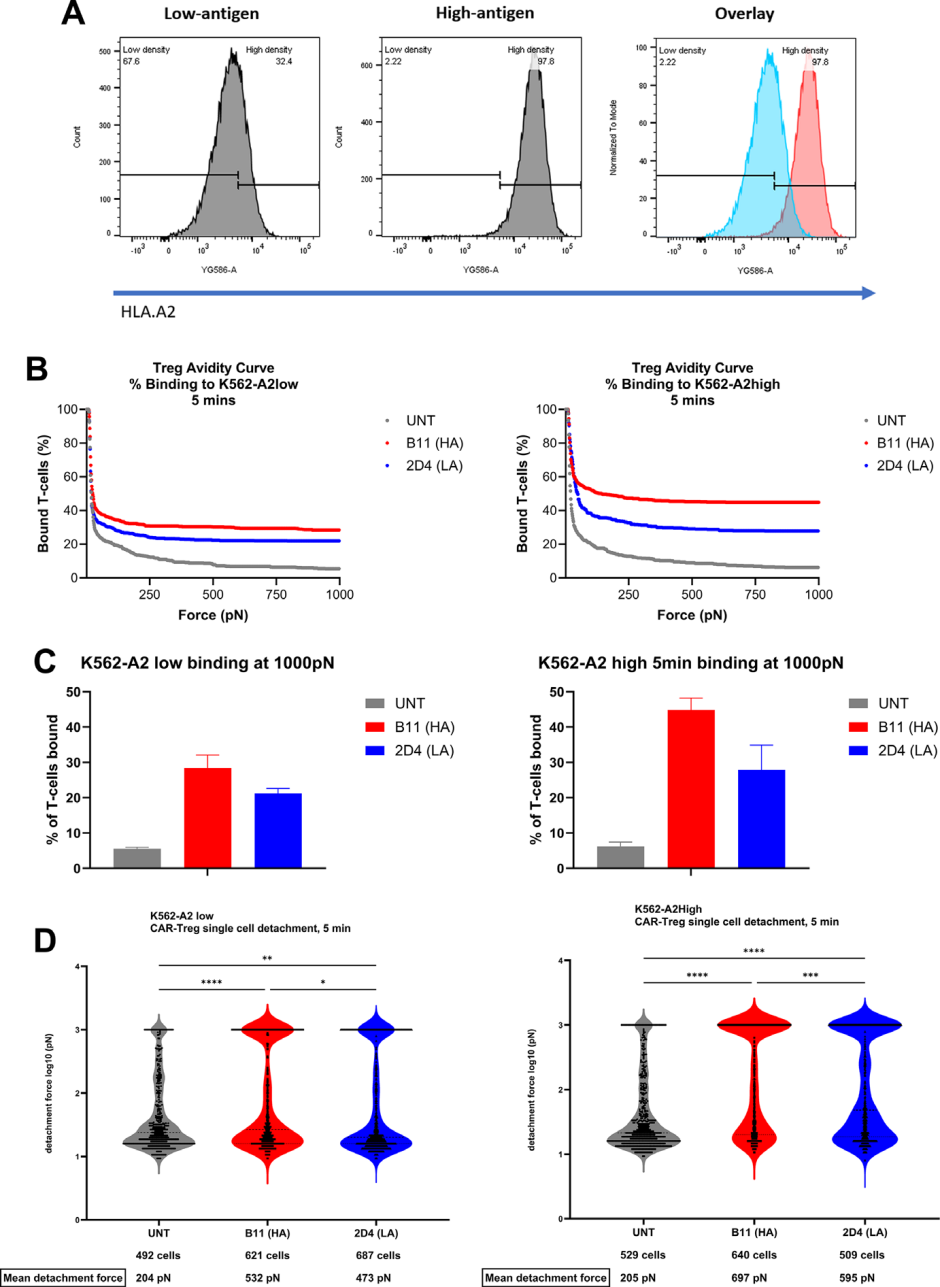
Avidity of different preparations of CAR‐Tregs in the presence of high and low antigen concentration. (A) HLA‐A2 expression levels on sorted SAL‐A2 target cells (measured 24 h before the avidity assays). (B) Avidity curves (Z‐movi assay) of Tregs incubated for 5 min with SAL‐A2 target cells expressing low and high densities of HLA‐A2, corresponding to two representative experiments. (C) Proportion of CAR‐Tregs that remained bound after applying a maximal detachment force of 1000 pN following 5 min of incubation with SAL‐A2 target cells expressing low and high HLA‐A2 densities. (D) Violin plots showing the total number of Tregs that detach from the SAL‐A2 target cells at the different forces (log10) applied. Bar plots in (B, C) display mean ± SD; Violin plots (D) represent individual data points with median and quartiles of the dataset. Statistical analyses were performed employing one‐way ANOVA with Tukey's multiple comparison tests. *p < 0.05, **p < 0.01, ***p < 0.001, ****p < 0.0001. UNT: untransduced Tregs; 2D4 (LA): low‐affinity CAR‐Tregs; B11 (HA): high‐affinity CAR‐Tregs.

To understand the mechanisms underpinning the superior functional properties of LA CAR‐Tregs, we first investigated the extent to which scFv affinity influences the overall strength of the interactions between CAR‐Treg and antigen‐presenting cells. For this purpose, we generated SAL‐A2 monolayers expressing high and low HLA‐A2 antigen density (Figure 6A). Analysis of avidity curves revealed that HA CAR‐Tregs displayed higher overall avidity than LA CAR‐Tregs, with the greatest divergence observed at high levels of HLA‐A2 expression (Figure 6B). Consistent with this, quantification of CAR‐Tregs remaining bound under maximal detachment force showed that the HA CAR‐Tregs maintained higher avidity at both densities of their target antigen. At low antigen density, 28.44% of HA CAR‐Tregs remained bound compared with 21.21% of LA CAR‐Tregs, while at high antigen density this increased to 44.89% versus 27.84%, respectively (Figure 6C). These results indicate that HA CAR‐Tregs exhibit higher avidity for HLA‐A2‐expressing cells than LA CAR‐Tregs, especially at high levels of target antigen density. Additionally, measuring the mean force required to detach CAR‐Tregs showed that HA CAR‐Tregs required higher forces to detach from monolayers expressing their target antigen, averaging 532 pN at low antigen density and 687 pN at high density, compared with 473 pN and 595 pN for LA CAR‐Tregs, respectively, indicating a longer duration of CAR and antigen engagement (Figure 6D). Altogether, these results demonstrate that, as compared with LA CAR‐Tregs, HA CAR‐Tregs exhibit higher avidity and a prolonged engagement between the CAR and the target cognate antigen.

### 2.7 The scFv Affinity Influences CAR Internalization and/or Recycling and Results in Differences in the Trogocytosis of Antigen Presenting Cells

**FIGURE 7:**
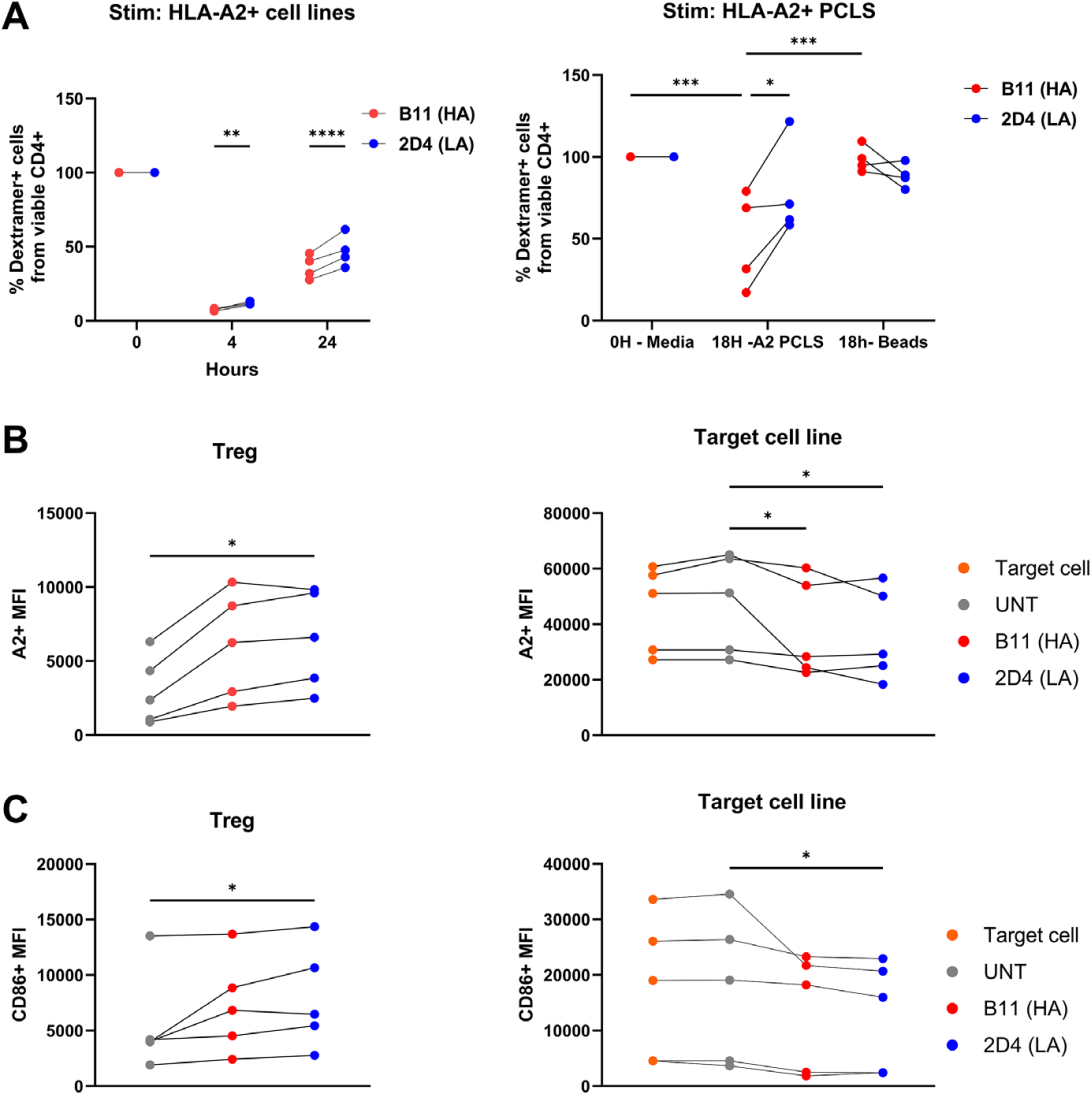
Impact of CAR affinity on the downregulation of CAR surface expression and trogocytosis. (A) Differences in the proportion of dextramer^+^ cells among total CD4+ T cells between high‐ and low‐affinity CAR‐Tregs at different time points following culture with HLA‐A2‐expressing K562 cells (left panel) and HLA‐A2^+^ PCLS (right panel; *n* = 4 for both experiments). (B) MFI of HLA‐A2 expressed on Tregs (left panel) or HLA‐A2 expressing K562 cells (right panel) after 4 h of co‐culture (*n* = 5). (C) MFI of CD86 expressed on Tregs (left panel) or HLA‐A2 expressing K562 cells (right panel) after 4 h of co‐culture (*n* = 5). For all panels, each dot represents an independent Treg donor. Statistical analyses were conducted using repeated measures two‐way ANOVA with Tukey's multiple comparison test (A), and nonparametric Friedman's test with Dunn's multiple comparison test (B, C). *p < 0.05, **p < 0.01, ***p < 0.001, ****p < 0.0001. UNT: untransduced Tregs; 2D4 (LA): low‐affinity CAR‐Tregs; B11 (HA): high‐affinity CAR‐Tregs; MFI: mean fluorescence intensity; PCLS: precision‐cut liver slices.

CAR engagement has been shown to cause target antigen stripping via trogocytosis [32] followed by progressive downregulation of the CAR expression [33, 34]. Tregs employ the same mechanism to nibble costimulatory molecules such as CD80 and CD86 from APCs, resulting in reduced APC immunogenicity [35, 36]. We investigated the impact of antigen engagement on CAR expression by co‐culturing CAR‐Tregs with HLA‐A2^+^ cell lines and PCLS and quantifying dextramer expression. After co‐culture with HLA‐A2^+^ cell lines, the percentage of CAR^+^ cells dropped for both HA and LA CAR‐Tregs, with HA CAR‐Tregs exhibiting a consistently greater downregulation of the CAR than LA CAR‐Tregs, both at 4 and 24 h. Similar findings were observed following culture with PCLS (Figure 7A). These data suggest that the stronger antigen engagement provided by the HA CAR may interfere with Treg function by reducing the overall surface expression of the CAR. To assess whether CAR affinity also influences the trogocytosis of APC‐expressed target antigen and costimulatory molecules, we co‐cultured CAR‐Tregs with HLA‐A2^+^ B‐LCLs and analyzed HLA‐A2 and CD86 expression on both Tregs and target cells (Table S5). Both CAR‐Treg products depleted HLA‐A2 from B‐LCLs more effectively than untransduced Tregs, but only LA CAR‐Tregs exhibited a significant increase in surface HLA‐A2 expression compared with untransduced Tregs (Figure 7B). LA CAR‐Tregs also more efficiently depleted CD86 (Figure 7C; Figures S8 and S9), indicating that CAR affinity and/or CAR surface expression regulate the capacity of Tregs to trogocytose APCs.

## 3 Discussion

Although CARs induce T cell activation by recapitulating some of the key downstream signaling responses elicited by TCRs [37], they differ from TCRs in that they do not form classical immunological synapses, and they exhibit drastically different kinetics, spatial organization, and sensitivity to antigen. TCRs are highly sensitive, allowing fine‐tuned responses to low antigen densities, whereas CARs require higher antigen densities and often exhibit tonic signaling due to constitutive receptor clustering [38]. The affinity of a TCR for its cognate peptide‐MHC (pMHC) typically ranges between 1 and 100 µM [39]. Notably, effector T cells engineered to express TCRs with pMHC affinities above this physiological range develop exhaustion and show poor in vivo persistence [40, 41, 42]. This has obvious implications for the therapeutic efficacy of CAR‐T cells, given that scFvs exhibit antigen affinities far exceeding those of TCRs. Some studies [20, 25, 26], although not others [27, 28], indicate that reducing the affinity of the CAR improves the cytotoxic functionality of CAR‐T cells. This has been attributed to the capacity of low‐affinity CAR‐Ts to engage and disengage with multiple tumor cells in a sequential manner, essentially becoming serial killers, in contrast to the much stronger engagement and poor dissociation of high‐affinity CAR‐T cells [43]. Furthermore, high‐affinity CAR‐T cells exhibit increased capacity to nibble membrane fragments from target cells (trogocytosis). This can result in reduced CAR‐T expansion, persistence, and efficacy, as a result of either CAR‐T fratricide (i.e., CAR‐T cells expressing antigens from the target cell being killed by other CAR‐Ts) or tumor escape (due to cancer cell antigen loss) [32, 45]. Finally, effector CAR‐T cells with high‐affinity CARs are more likely to develop exhaustion following chronic stimulation than T cells expressing low‐affinity CARs [44]. In contrast to the extensive published literature describing the influence of CAR affinity on effector T cell function, the extent to which this impacts upon CAR‐Treg phenotype, stability, and function remains unclear.

Our study shows that reducing the affinity of the CAR scFv by approximately 60‐fold (from *K*_D_ = 1–45 nM) significantly increases the activation level of CAR‐Tregs following antigenic stimulation, resulting in better tissue infiltration and more powerful immunosuppressive effects, both in vitro and in vivo. The biophysical properties of CAR binding depend not only on scFv affinity, but also on avidity and target antigen density [45]. Our results demonstrate that LA CAR‐Tregs exhibit lower avidity than HA CAR‐Tregs, which could facilitate serial triggering and enhanced activation via weaker and shorter‐duration ligand–receptor interactions, leaving more HLA‐A2 molecules available for antigen recognition. Conversely, the higher avidity of HA CAR‐Tregs could result in stronger receptor–ligand interactions with their target cells, followed by rapid CAR ubiquitination, internalization, and lysosomal degradation, ultimately reducing CAR surface expression and CAR‐Treg activation [46, 47]. The latter is supported by the results of our co‐culture experiments employing HLA‐A2^+^ human PCLS and tissue infiltration in mouse PCLS. Although stronger CAR–antigen interactions in HA CAR‐Tregs could potentially promote enhanced tissue retention, the reduced proportion of GFP^+^ cells following homogenization of PCLS, together with the lower absolute number of CAR^+^ cells, indicates that enhanced adherence is unlikely to account for these findings. Furthermore, the increased capacity of LA CAR‐Tregs to reduce the immunogenicity of APCs by nibbling CD86 molecules from their surface membrane constitutes an additional mechanism to account for their more powerful immunosuppressive properties. We propose that a combination of these mechanisms accounts for the longer persistence of LA CAR‐Tregs in vivo, reduced GVHD severity, and improved recipient survival, as compared with HA CAR‐Tregs.

It is important to acknowledge that the functionality of a CAR‐Treg product is dependent on many factors other than the scFv affinity, including the overall CAR design, target antigen, and Treg phenotype and stability. This explains the diverging results recently reported by Cochrane et al. [48], who compared high and low‐affinity anti‐CD19 CAR‐Tregs and observed that those with high‐affinity lost their regulatory phenotype and acquired a pro‐inflammatory profile. Therefore, the generalizability of our results to other CARs and different target antigens will need to be demonstrated experimentally. Likewise, our study has not identified the specific level of CAR affinity providing the most optimal functionality. Further studies under prolonged antigen stimulation will be required to define the long‐term impact of affinity tuning on CAR‐Treg stability and receptor kinetics in clinically relevant settings. Considering that the sensitivity of CARs is 10–100‐fold lower than that of TCRs [49, 50], we hypothesize that for each level of target antigen density, there will be a specific CAR affinity threshold below which no further functional advantage will be obtained. We anticipate that this will need to be determined for each CAR‐antigen pair [49].

In summary, we conclude that by decreasing the affinity of the scFvs, the function of anti‐HLA‐A2 CAR‐Tregs can be enhanced, both in vitro and in vivo. This results in more robust activation, more powerful suppressive properties, better tissue infiltration, and more prolonged in vivo persistence. Our findings suggesting that affinity modulation is crucial for optimizing the efficacy of CAR‐Treg therapies have important implications for ongoing and future clinical trials employing anti‐HLA‐A2 CAR‐Tregs in organ transplantation. Affinity‐tuning of CAR‐Tregs should also be considered when selecting CARs targeting other antigens and when benchmarking different CAR‐Treg products.

## 4 Materials and Methods

### 4.1 Generating Affinity‐Tuned CAR DNA

We selected three VL scFv domains with differing antigen affinities, and these HLA‐A2 light chain sequences were previously described in a paper by Watkins et al. [51]. They were determined by titration of purified single‐chain variable fragments on HLA‐A2‐positive platelets. The heavy chain domain remained the same across all three constructs. The scFv regions were incorporated into a CAR backbone containing a CD8 leader, CD28 costimulatory domain, C‐Myc tag, and CD3ζ signaling domain. The finalized CAR constructs were cloned into lentiviral plasmids that contained an eGFP gene fused to the CD3ζ signaling domain and an ampicillin resistance gene (Figure 1A). The high‐affinity (HA) CAR was named B11, the moderate‐affinity (MA) CAR was named B10, and the low‐affinity (LA) CAR was named 2D4.

### 4.2 Human Blood Samples

Leukocyte‐enriched blood cones from the National Blood Service were used (Research Ethics Committee [REC] approval number: REC Number15/NS/0062).

### 4.3 Treg Isolation and Expansion

HLA‐A2 CAR‐Tregs with different affinities were manufactured using HLA‐A2–negative PBMCs obtained from healthy donor leukocyte cones. For Treg isolation and sorting, we initially enriched for CD4^+^ cells using Rosette‐Sep human CD4^+^ T cell enrichment cocktail (StemCell; cat. 15022) and subsequently performed CD4^+^CD25^+^ cell selection with anti‐CD25 microbeads (Miltenyi Biotec; cat. 130‐092‐983). Tregs were then sorted using a Highway 1 flow cytometer based on CD4^+^CD25^+^CD127^−^ surface expression. Following sorting, cells were washed and resuspended in X‐VIVO (Lonza) supplemented with 5% human AB serum (Sigma‐Aldrich, H4522) along with 1000 IU/mL IL‐2 (Proleukin, Novartis). Cells were seeded at 1 × 10^6^ cells/mL in a 24‐well plate and were stimulated with anti‐CD3/CD28 Dynabeads in a 1:1 ratio. 2000 IU/mL 7 of IL‐2 was added every 2 days. Cells were restimulated at day 9.

### 4.4 CAR Transduction

Tregs underwent lentiviral transduction using retronectin‐coated plates (Takara‐Bio, T100B) with second‐generation affinity‐tuned CARs specific for HLA‐A2. Transduction was performed on day 4 postsorting. Posttransduction, 1000 IU of IL‐2 was added at 24 h, and transduction efficiency was assessed at 72 h by measuring the percentage of GFP+ cells via flow cytometry. To confirm the expression of the anti‐HLA.A2 CAR, transduced Tregs were stained with an APC‐labelled HLA.A*0201 dextramer (Immudex‐cat. WB3307). We phenotypically characterized all CAR‐Treg products on days 3 and 10 after transduction.

### 4.5 Flow Cytometric Surface and Intracellular Staining

Staining buffer (PBS with 2.5% bovine serum albumin) was used for all the flow cytometric staining washes. Cells were stained with live/dead viability dye in PBS (10 min, ice, dark) and incubated with surface antibody master mixes (25 min, 4°C, dark). Following fixation, intracellular staining was performed using fixation/permeabilization buffers (ThermoFisher, cat: 00‐5123‐43, 00‐5223‐56, and 00‐8333‐56) according to the manufacturer's instructions, with antibody incubation for 30 min at room temperature in the dark. After final washes and fixation, samples were stored at 4°C and acquired within 24 h on a BD FACS Fortessa X‐20. Data were analyzed using FlowJo v10.8.

### 4.6 In Vitro T Cell Functional Assays

We conducted Treg activation, suppression, and stability assays to compare the functional properties of the generated CAR‐Treg products. For the activation‐induced marker assay to identify alloreactive Treg clones, Treg products were stimulated with 40‐Gy irradiated EBV‐transformed allogeneic B‐lymphoblastic cell lines (B‐LCL) cells that were either HLA‐A2^−^ (BM21; HLA‐A2^−^DR11^+^) or HLA‐A2^+^ (SPO; HLA‐A2^+^DR11^+^). Tregs were harvested and stained after 3, 6, and 24 h to examine the expression of activation markers. To assess the suppressive capacity of CAR‐Tregs, autologous T effector cells were stained with APC proliferation dye and then were stimulated with irradiated 1:1 CTV‐stained HLA‐A2+ B‐LCLs, in the presence of LA and HA CAR‐Tregs at 1:10 and 1:25 ratios. After 5 days of co‐culture, T effector proliferation was assessed in Fortessa, and the percentage of suppression was calculated as 100 – (Percentage proliferation observed in the presence of Tregs/Percentage proliferation observed with T effector + B cell stimulation) × 100. For the stability assays, we stimulated Tregs either through their TCR with CD3/CD28 beads or through their CAR by culturing them with A2‐expressing single antigen cell lines (SAL‐A2), at a ratio of 1:1, in the presence of 100U low‐dose IL‐2 for 5 days. We assessed the phenotype of the cells at the end of 5 days. To determine the trogocytosis of HLA‐A2 from stimulant cells of A2 expressing B‐LCLs, we co‐cultured untransduced (UNT) and CAR‐Tregs with B‐LCLs at a ratio of 1 Treg:1 stimulant and assessed HLA‐A2 expression on cell lines and Tregs following 4 h of co‐culture.

### 4.7 Ex Vivo CAR‐Treg Activation Using Precision‐Cut Liver Slices

#### 4.7.1 Mouse Liver Tissue

Transgenic homozygous Tg (HLA‐A2.1)1Enge mice, which express high levels of the human class I MHC antigen HLA‐A2.1, were used in these experiments (The Jackson Laboratory, C57BL/6‐*Mcph1^Tg(HLA‐A2.1)1Enge^*/J). All animals were housed under specific pathogen‐free conditions, and all experimental procedures were conducted in accordance with institutional, national, and ethical regulations (PPL70/09066).

#### 4.7.2 Human Liver Tissue

Human liver tissue was obtained from hepatectomies performed at King's College Hospital. After surgical resection, liver tissue samples from the specimen were obtained in an area unaffected by and distant from the lesion for which surgical resection was intended. The liver tissue was immediately put in ice‐cold Belzer UW Cold Storage Solution. All participants signed their informed consent before the donation of liver tissue (REC approval number: 15/NS/0062).

#### 4.7.3 Production and Culture of Precision‐Cut Liver Slices

Liver tissue was punched with a disposable 3–5 mm biopsy puncher, and then the cores were submerged in low‐melting agarose. After the agarose in sat, the cores were sliced using a Leica VT1200S vibrating blade microtome (Leica Biosystems; speed: 1.5 mm/s, amplitude: 3 mm, step size: 250 µm), while being submerged in cold PBS (Gibco). Using a P1000 pipette with a cut tip, PCLS were transferred into a 24‐well plate with 8.0 µm high‐density PET membrane inserts and with preheated WME medium (Gibco), supplemented with 1% Ultraglutamine I (Lonza) and 1% Penicillin‐Streptomycin (Life Technologies), insulin‐transferrin‐selenium, 2% fetal bovine serum, and 100 nM of dexamethasone. The slices were cultured on an orbital shaker (Infors Celltron) at a speed of 80 rpm. PCLS were cultured at 37°C, and Tregs were added 2 h after to the slices. After 18 h co‐culture, precision cut liver slices (PCLS) were processed into single‐cell suspensions, and dissociated tissue was filtered through 70‐µm strainers, washed to remove debris and aggregates, and stained. Immune‐cell infiltration was quantified by flow cytometry and anti‐GFP immunohistochemistry staining. Experiments were performed using independent Treg donors and independent liver slices.

### 4.8 Assessing Cell Avidity Using Z‐movi

SAL‐A2 cells expressing HLA‐A2 at high and low antigen densities were generated to be used as monolayers. Five days before Z‐movi experiments, 30 × 10^6^ cells were fluorescently sorted based on HLA‐A2 expression using PE anti‐human HLA‐A2 antibody. These cells were used within a week postsorting to prevent normalization of HLA‐A2 expression. Before performing assays, the monolayers had 60%–80% confluency and 85% viability, while Tregs maintained 80% viability, a stable phenotype, and 70% transduction efficacy. For avidity assays, chips were prepared with Concanavalin A coating for the SAL‐A2 monolayer. Tregs were co‐incubated with the SAL‐A2 monolayer for varying times (2, 5, and 10 min) and exposed to acoustic force via Z‐movi to assess immune synapse strength. Brightfield imaging was used to evaluate monolayer confluency, and 200–400 cells per run were analyzed. Data analysis using Ocean software provided single‐cell analysis and avidity curves, which were further processed in GraphPad Prism to plot the percentage of Tregs bound to HLA‐A2‐expressing target cell lines upon application of increasing force.

### 4.9 In Vivo Xenogeneic GvHD Model

Eight‐week‐old female NOD SCID gamma mice (Charles River) received 1.5‐Gy irradiation and an intravenous infusion of 10 × 10^6^ HLA‐A2^+^ allogenic donor PBMCs, alone or in combination with 5 × 10^6^ LA and HA CAR‐Treg cells (1:2 ratio). Progression of GvHD was monitored daily by following GvHD clinical grading criteria based on Cooke et al.’s assessment criteria [52], and we tested for tail blood for engraftment every 7 days. Animals were maintained under sterile conditions, and all procedures were performed following all legal, ethical, and institutional requirements (PPL70/09066).

### 4.10 Statistical Analyses

Statistical analyses were conducted using GraphPad Prism 9.0 software. Specific statistical tests performed for each experiment are detailed in the corresponding figure legends. For comparisons involving more than two groups, one‐way or two‐way ANOVA with Tukey's post hoc correction for pairwise comparisons was applied. Statistical significance was defined as *p* < 0.05, and the following thresholds were used to determine significance: *p < 0.05, **p < 0.01, ***p < 0.001, and ****p < 0.0001. All results are expressed as mean ± SD unless otherwise specified in the figure legends.

## Author Contributions

A.S.‐F. and M.M.‐L. conceptualized and supervised the project. M.E., E.L., E.K., J.T.‐Y., and P.R. helped with the methodology. A.S.K., M.E., A.P., Q.B., M.S., and H.M. performed the experiments and analyzed the data. A.S.K. and A.S.‐F. wrote the original draft, and all authors reviewed and approved the final manuscript.

## Ethics Statement

Leukocyte‐enriched blood cones from the National Blood Service were used in accordance with protocols approved by the Research Ethics Committee [REC] approval number: REC Number15/NS/0062. All experimental procedures were conducted under sterile conditions per Home Office regulations. The animal study was reviewed and approved by the Animal Welfare and Ethical Review Body of King's College London, and all procedures were performed following all legal, ethical, and institutional requirements (PPL70/09066).

## Conflicts of Interest

A.S.‐F. and M.M.‐L. are founders of Quell Therapeutics, and M.M‐L is also an employee of Quell Therapeutics. A.S.‐F. holds equity and stocks in Quell Therapeutics. The remaining authors declare no conflicts of interest.

## Supporting information




**Supporting File**: eji70226‐sup‐0001‐SuppMat.pdf.

## Data Availability

The data that support the findings of this study are available from the corresponding author upon reasonable request.

## References

[1] G. Plitas and A. Y. Rudensky , “Regulatory T Cells: Differentiation and Function,” Cancer Immunology Research 4 (2016): 721–725, 10.1158/2326-6066.CIR-16-0193. 27590281 PMC5026325

[2] B. Akkaya , Y. Oya , M. Akkaya , et al., “Regulatory T Cells Mediate Specific Suppression by Depleting Peptide–MHC Class II From Dendritic Cells,” Nature Immunology 20 (2019): 218–231, 10.1038/s41590-018-0280-2. 30643268 PMC6402611

[3] K. Wing , Y. Onishi , P. Prieto‐Martin , et al., “CTLA‐4 Control Over Foxp3^+^ Regulatory T Cell Function,” Science 322 (1979): 271–275, 10.1126/science.1160062. 18845758

[4] A. Chaudhry , R. M. Samstein , P. Treuting , et al., “Interleukin‐10 Signaling in Regulatory T Cells Is Required for Suppression of Th17 Cell‐Mediated Inflammation,” Immunity 34 (2011): 566–578, 10.1016/j.immuni.2011.03.018. 21511185 PMC3088485

[5] M. Romano , G. Fanelli , C. J. Albany , G. Giganti , and G. Lombardi , “Past, Present, and Future of Regulatory T Cell Therapy in Transplantation and Autoimmunity,” Frontiers in Immunology 10 (2019): 43, 10.3389/fimmu.2019.00043. PMC6371029 30804926

[6] C. G. Brunstein , J. S. Miller , Q. Cao , et al., “Infusion of Ex Vivo Expanded T Regulatory Cells in Adults Transplanted With Umbilical Cord Blood: Safety Profile and Detection Kinetics,” Blood 117 (2011): 1061–1070, 10.1182/blood-2010-07-293795. 20952687 PMC3035067

[7] M. F. Martelli , M. Di Ianni , L. Ruggeri , et al., “HLA‐haploidentical Transplantation With Regulatory and Conventional T‐cell Adoptive Immunotherapy Prevents Acute Leukemia Relapse,” Blood 124 (2014): 638–644, 10.1182/blood-2014-03-564401. 24923299

[8] P. Trzonkowski , A. Dukat‐Mazurek , M. Bieniaszewska , et al., “Treatment of Graft‐Versus‐Host Disease With Naturally Occurring T Regulatory Cells,” Biodrugs 27 (2013): 605–614, 10.1007/s40259-013-0050-5. 23813436 PMC3832760

[9] J. A. Bluestone , J. H. Buckner , M. Fitch , et al., “Type 1 Diabetes Immunotherapy Using Polyclonal Regulatory T Cells,” Science Translational Medicine 7, no. 315 (2015): 315ra189, 10.1126/scitranslmed.aad4134. PMC4729454 26606968

[10] A. Sánchez‐Fueyo , G. Whitehouse , N. Grageda , et al., “Applicability, Safety, and Biological Activity of Regulatory T Cell Therapy in Liver Transplantation,” American Journal of Transplantation 20 (2020): 1125–1136, 10.1111/ajt.15700. 31715056 PMC7154724

[11] C. S. Bader , A. Pavlova , R. Lowsky , et al., “Single‐center Randomized Trial of T‐reg Graft Alone vs T‐reg Graft plus Tacrolimus for the Prevention of Acute GVHD,” Blood Advances 8 (2024): 1105–1115, 10.1182/bloodadvances.2023011625. 38091578 PMC10907400

[12] Q. Tang , K. J. Henriksen , M. Bi , et al., “In Vitro–Expanded Antigen‐Specific Regulatory T Cells Suppress Autoimmune Diabetes,” The Journal of Experimental Medicine 199 (2004): 1455–1465, 10.1084/jem.20040139. 15184499 PMC2211775

[13] J. Y.‐S. Tsang , Y. Tanriver , S. Jiang , et al., “Conferring Indirect Allospecificity on CD4+CD25+ Tregs by TCR Gene Transfer Favors Transplantation Tolerance in Mice,” Journal of Clinical Investigation 118 (2008): 3619–3628, 10.1172/JCI33185. 18846251 PMC2564608

[14] P. Sagoo , N. Ali , G. Garg , F. O. Nestle , R. I. Lechler , and G. Lombardi , “Human Regulatory T Cells With Alloantigen Specificity Are More Potent Inhibitors of Alloimmune Skin Graft Damage Than Polyclonal Regulatory T Cells,” Science Translational Medicine 3, no. 83 (2011): 83ra42, 10.1126/scitranslmed.3002076. PMC3776382 21593402

[15] A. L. Putnam , N. Safinia , A. Medvec , et al., “Clinical Grade Manufacturing of Human Alloantigen‐Reactive Regulatory T Cells for Use in Transplantation,” American Journal of Transplantation 13 (2013): 3010–3020, 10.1111/ajt.12433. 24102808 PMC4161737

[16] A. S. Kurt , P. Ruiz , E. Landmann , et al., “Conferring Alloantigen Specificity to Regulatory T Cells: A Comparative Analysis of Cell Preparations Undergoing Clinical Development in Transplantation,” American Journal of Transplantation 25 (2025): 38–47, 10.1016/j.ajt.2024.09.009. 39299674

[17] S. E. James , P. D. Greenberg , M. C. Jensen , et al., “Antigen Sensitivity of CD22‐Specific Chimeric TCR Is Modulated by Target Epitope Distance From the Cell Membrane,” The Journal of Immunology 180 (2008): 7028–7038, 10.4049/jimmunol.180.10.7028. 18453625 PMC2585549

[18] M. Hudecek , D. Sommermeyer , P. L. Kosasih , et al., “The Nonsignaling Extracellular Spacer Domain of Chimeric Antigen Receptors Is Decisive for in Vivo Antitumor Activity,” Cancer Immunology Research 3 (2015): 125–135, 10.1158/2326-6066.CIR-14-0127. 25212991 PMC4692801

[19] J. S. Bridgeman , R. E. Hawkins , S. Bagley , M. Blaylock , M. Holland , and D. E. Gilham , “The Optimal Antigen Response of Chimeric Antigen Receptors Harboring the CD3ζ Transmembrane Domain Is Dependent Upon Incorporation of the Receptor Into the Endogenous TCR/CD3 Complex,” The Journal of Immunology 184 (2010): 6938–6949, 10.4049/jimmunol.0901766. 20483753

[20] L. Labanieh , R. G. Majzner , and C. L. Mackall , “Programming CAR‐T Cells to Kill Cancer,” Nature Biomedical Engineering 2 (2018): 377–391, 10.1038/s41551-018-0235-9. 31011197

[21] V. D. Fedorov , M. Themeli , and M. Sadelain , “PD‐1– and CTLA‐4–Based Inhibitory Chimeric Antigen Receptors (iCARs) Divert Off‐Target Immunotherapy Responses,” Science Translational Medicine 5, no. 215 (2013): 215ra172, 10.1126/scitranslmed.3006597. PMC4238416 24337479

[22] N. A. J. Dawson , I. Rosado‐Sánchez , G. E. Novakovsky , et al., “Functional Effects of Chimeric Antigen Receptor Co‐Receptor Signaling Domains in Human Regulatory T Cells,” Science Translational Medicine 12, no. 557 (2020): eaaz3866, 10.1126/scitranslmed.aaz3866. 32817364

[23] C. Zhang , J. Liu , J. F. Zhong , and X. Zhang , “Engineering CAR‐T Cells,” Biomarker Research 5 (2017): 22, 10.1186/s40364-017-0102-y. 28652918 PMC5482931

[24] E. R. Vander Mause , D. Atanackovic , C. S. Lim , and T. Luetkens , “Roadmap to Affinity‐Tuned Antibodies for Enhanced Chimeric Antigen Receptor T Cell Function and Selectivity,” Trends in Biotechnology 40 (2022): 875–890, 10.1016/j.tibtech.2021.12.009. 35078657

[25] H. G. Caruso , L. V. Hurton , A. Najjar , et al., “Tuning Sensitivity of CAR to EGFR Density Limits Recognition of Normal Tissue While Maintaining Potent Antitumor Activity,” Cancer Research 75 (2015): 3505–3518, 10.1158/0008-5472.CAN-15-0139. 26330164 PMC4624228

[26] S. Ghorashian , A. M. Kramer , S. Onuoha , et al., “Enhanced CAR T Cell Expansion and Prolonged Persistence in Pediatric Patients With ALL Treated With a Low‐affinity CD19 CAR,” Nature Medicine 25 (2019): 1408–1414, 10.1038/s41591-019-0549-5. 31477906

[27] M. Hudecek , M.‐T. Lupo‐Stanghellini , P. L. Kosasih , et al., “Receptor Affinity and Extracellular Domain Modifications Affect Tumor Recognition by ROR1‐Specific Chimeric Antigen Receptor T Cells,” Clinical Cancer Research 19 (2013): 3153–3164, 10.1158/1078-0432.CCR-13-0330. 23620405 PMC3804130

[28] R. C. Lynn , Y. Feng , K. Schutsky , et al., “High‐affinity FRβ‐Specific CAR T Cells Eradicate AML and Normal Myeloid Lineage Without HSC Toxicity,” Leukemia 30 (2016): 1355–1364, 10.1038/leu.2016.35. 26898190 PMC4889499

[29] F. Noyan , K. Zimmermann , M. Hardtke‐Wolenski , et al., “Prevention of Allograft Rejection by Use of Regulatory T Cells with an MHC‐Specific Chimeric Antigen Receptor,” American Journal of Transplantation 17 (2017): 917–930, 10.1111/ajt.14175. 27997080

[30] K. G. MacDonald , R. E. Hoeppli , Q. Huang , et al., “Alloantigen‐Specific Regulatory T Cells Generated With a Chimeric Antigen Receptor,” Journal of Clinical Investigation 126 (2016): 1413–1424, 10.1172/JCI82771. 26999600 PMC4811124

[31] D. A. Boardman , C. Philippeos , G. O. Fruhwirth , et al., “Expression of a Chimeric Antigen Receptor Specific for Donor HLA Class I Enhances the Potency of Human Regulatory T Cells in Preventing Human Skin Transplant Rejection,” American Journal of Transplantation 17 (2017): 931–943, 10.1111/ajt.14185. 28027623

[32] M. L. Olson , E. R. V. Mause , S. V. Radhakrishnan , et al., “Low‐Affinity CAR T Cells Exhibit Reduced Trogocytosis, Preventing Rapid Antigen Loss, and Increasing CAR T Cell Expansion,” Leukemia 36 (2022): 1943–1946, 10.1038/s41375-022-01585-2. 35490197 PMC9252916

[33] R. Greenman , Y. Pizem , M. Haus‐Cohen , et al., “Phenotypic Models of CAR T‐Cell Activation Elucidate the Pivotal Regulatory Role of CAR Downmodulation,” Molecular Cancer Therapeutics 20 (2021): 946–957, 10.1158/1535-7163.MCT-19-1110. 33649103

[34] N. Trendel , P. Kruger , S. Gaglione , et al., “Perfect Adaptation of CD8^+^ T Cell Responses to Constant Antigen Input Over a Wide Range of Affinities is Overcome by Costimulation,” Science Signaling 14 (2021): eaay9363, 10.1126/scisignal.aay9363. 34855472 PMC7615691

[35] M. Tekguc , J. B. Wing , M. Osaki , J. Long , and S. Sakaguchi , “Treg‐Expressed CTLA‐4 Depletes CD80/CD86 by Trogocytosis, Releasing Free PD‐L1 on Antigen‐Presenting Cells,” Proceedings of the National Academy of Sciences 118, no. 30 (2021): e2023739118, 10.1073/pnas.2023739118. PMC8325248 34301886

[36] K. Miyake and H. Karasuyama , “The Role of Trogocytosis in the Modulation of Immune Cell Functions,” Cells 10 (2021): 1255, 10.3390/cells10051255. 34069602 PMC8161413

[37] A. J. Davenport , R. S. Cross , K. A. Watson , et al., “Chimeric Antigen Receptor T Cells Form Nonclassical and Potent Immune Synapses Driving Rapid Cytotoxicity,” Proceedings of the National Academy of Sciences 115, no. 9 (2018), E2068–E2076, 10.1073/pnas.1716266115. PMC5834689 29440406

[38] A. I. Salter , R. G. Ivey , J. J. Kennedy , et al., “Phosphoproteomic Analysis of Chimeric Antigen Receptor Signaling Reveals Kinetic and Quantitative Differences That Affect Cell Function,” Science Signaling 11, no. 544 (2018): eaat6753, 10.1126/scisignal.aat6753. 30131370 PMC6186424

[39] C. Lamarche , K. Ward‐Hartstonge , T. Mi , et al., “Tonic‐signaling Chimeric Antigen Receptors Drive human Regulatory T Cell Exhaustion,” Proceedings of the National Academy of Sciences 120, no. 14 (2023): e2219086120, 10.1073/pnas.2219086120. PMC10083618 36972454

[40] S. Park , E. Shevlin , Y. Vedvyas , et al., “Micromolar Affinity CAR T Cells to ICAM‐1 Achieves Rapid Tumor Elimination While Avoiding Systemic Toxicity,” Scientific Reports 7 (2017): 14366, 10.1038/s41598-017-14749-3. 29085043 PMC5662687

[41] M. L. Sprouse , I. Shevchenko , M. A. Scavuzzo , et al., “Cutting Edge: Low‐Affinity TCRs Support Regulatory T Cell Function in Autoimmunity,” The Journal of Immunology 200 (2018): 909–914, 10.4049/jimmunol.1700156. 29282307 PMC5962277

[42] M. Chmielewski , A. Hombach , C. Heuser , G. P. Adams , and H. T. Abken , “T Cell Activation by Antibody‐Like Immunoreceptors: Increase in Affinity of the Single‐Chain Fragment Domain Above Threshold Does Not Increase T Cell Activation Against Antigen‐Positive Target Cells but Decreases Selectivity,” The Journal of Immunology 173 (2004): 7647–7653, 10.4049/jimmunol.173.12.7647. 15585893

[43] C. H. June , “Serial Killers and Mass Murderers: Engineered T Cells Are up to the Task,” Cancer Immunology Research 3 (2015): 470–472, 10.1158/2326-6066.CIR-15-0075. 25941357

[44] E. Drent , M. Themeli , R. Poels , et al., “A Rational Strategy for Reducing On‐Target Off‐Tumor Effects of CD38‐Chimeric Antigen Receptors by Affinity Optimization,” Molecular Therapy 25 (2017): 1946–1958, 10.1016/j.ymthe.2017.04.024. 28506593 PMC5542711

[45] R. Mao , W. Kong , and Y. He , “The Affinity of Antigen‐Binding Domain on the Antitumor Efficacy of CAR T Cells: Moderate Is Better,” Frontiers in Immunology 13 (2022): 1032403, 10.3389/fimmu.2022.1032403. 36325345 PMC9618871

[46] W. Li , S. Qiu , J. Chen , et al., “Chimeric Antigen Receptor Designed to Prevent Ubiquitination and Downregulation Showed Durable Antitumor Efficacy,” Immunity 53 (2020): 456–470.e6, 10.1016/j.immuni.2020.07.011. 32758419

[47] C. Liu , T. Qi , J. J. Milner , Y. Lu , and Y. Cao , “Speed and Location Both Matter: Antigen Stimulus Dynamics Controls CAR‐T Cell Response,” Frontiers in Immunology 12 (2021): 748768, 10.3389/fimmu.2021.748768. 34691062 PMC8531752

[48] R. W. Cochrane , R. A. Robino , B. Granger , et al., “High‐affinity Chimeric Antigen Receptor Signaling Induces an Inflammatory Program in Human Regulatory T Cells,” Molecular Therapy—Methods & Clinical Development 32 (2024): 101385, 10.1016/j.omtm.2024.101385. 39687729 PMC11647616

[49] D. A. Schmid , M. B. Irving , V. Posevitz , et al., “Evidence for a TCR Affinity Threshold Delimiting Maximal CD8 T Cell Function,” The Journal of Immunology 184 (2010): 4936–4946, 10.4049/jimmunol.1000173. 20351194

[50] D. T. Harris , M. V. Hager , S. N. Smith , et al., “Comparison of T Cell Activities Mediated by Human TCRs and CARs That Use the Same Recognition Domains,” The Journal of Immunology 200 (2018): 1088–1100, 10.4049/jimmunol.1700236. 29288199 PMC5780198

[51] N. A. Watkins , T. R. Dafforn , M. Kuijpers , et al., “Molecular Studies of Anti‐HLA‐A2 Using Light‐Chain Shuffling: A Structural Model for HLA Antibody Binding,” Tissue Antigens 63 (2004): 345–354, 10.1111/j.0001-2815.2004.00194.x. 15009806

[52] K. Cooke , L. Kobzik , T. Martin , et al., “An Experimental Model of Idiopathic Pneumonia Syndrome After Bone Marrow Transplantation: I. The Roles of Minor H Antigens and Endotoxin,” Blood 88 (1996): 3230–3239, 10.1182/blood.V88.8.3230.bloodjournal8883230. 8963063

