# Supplementary material for: Tuning the Affinity of Chimeric Antigen Receptors Enhances the Function of Human Engineered Regulatory T Cells
Source: Eur J Immunol. 2026 Jun 17;56(6):e70226. doi: 10.1002/eji.70226 (PMC13273929; doi:10.1002/eji.70226)
Supplement: Supplementary file 1 — Supporting File: eji70226‐sup‐0001‐SuppMat.pdf. [file EJI-56-e70226-s001.pdf]

Restriction enzymes
CD8 LEADER
HLA-A2 scFv VH domain
HLA-A2 scFv VL domain
CD28 extracellular domain
C-Myc-tag
CD28 transmembrane domain
CD28/CD3z signalling domain

B11 High-affinity:

GGATCCACCGCCATGGCCCTGCCCGTGACCGCCCTGCTGCTGCCCCTGGCCCTGCTGCTGCACGCCG
CCCGGCCTCAGGTGCAGCTGGTGCAGAGCGGCGGCGGCGTGGTGCAGCCCGGAGGCAGCCTGAG
GGTGAGCTGCGCCGCCAGCGGCGTGACCCTGAGCGACTACGGCATGCACTGGGTGCGGCAGGCTC
CCGGCAAGGGCCTGGAGTGGATGGCCTTCATCCGGAACGACGGCAGCGACAAGTACTACGCCGAC
AGCGTGAAGGGCCGGTTCACCATCAGCCGGGACAACAGCAAGAAGACCGTGAGCCTGCAGATGAG
CAGCCTGCGGGCTGAGGACACCGCCGTGTACTACTGCGCCAAGAACGGCGAGAGCGGCCCTCTGG
ACTACTGGTACTTCGACCTGTGGGGCAGGGGAACCCTGGTGACCGTGAGCAGCGGCGGCGGAGGC
AGCGGTGGCGGAGGCAGCGGCGGAGGCGGTAGCGACGTGGTGATGACCCAGAGCCCCAGCAGCC
TGAGCGCCAGCGTGGGCGACCGGGTGACCATCACCTGCCAGGCCAGCCAGGACATCAGCAACTACC
TGAACTGGTACCAGCAGAAGCCCGGCAAGGCCCCTAAGCTGCTGATCTACGACGCCAGCAACCTGG
AGACCGGCGTGCCAAGCCGGTTCAGCGGCAGCGGAAGCGGCACCGACTTCACCTTCACCATCAGCA
GCCTGCAGCCTGAGGACATCGCCACCTACTACTGCCAGCAGTACGACAACCTGCCTCCCACCTTCGG
CGGAGGCACCAAGCTGACCGTGCTGGGCGCGGCCGCCATCGAGGTGGAGCAGAAGCTGATCAGCG
AGGAGGACCTGCTGGACAACGAGAAGAGCAACGGCACCATCATCCACGTGAAGGGCAAGCACCTG
TGCCCCAGCCCCCTGTTCCCCGGCCCCAGCAAGCCCTTCTGGGTGCTGGTGGTGGTGGGCGGCGTG
CTGGCCTGCTACAGCCTGCTGGTGACCGTGGCCTTCATCATCTTCTGGGTGCGGAGCAAGCGGAGCC
GGCTGCTGCACAGCGACTACATGAACATGACCCCCCGGCGGCCTGGGCCCACCCGCAAGCATTACC
AGCCCTATGCCCCACCACGCGACTTCGCAGCCTATCGCTCCAGAGTGAAGTTCAGCAGGAGCGCAG
AcgCCCCCGCGTACCAGCAGGGCCAGAACCAGCTCTATAACGAGCTCAATCTAGGACGAAGAGAGG
AGTACGATGTTTTGGACAAGAGACGTGGCCGGGACCCTGAGATGGGGGGAAAGCCGAGAAGGAA
GAACCCTCAGGAAGGCCTGTACAATGAACTGCAGAAAGATAAGATGGCGGAGGCCTACAGTGAGA
TTGGGATGAAAGGCGAGCGCCGGAGGGGCAAGGGGCACGATGGCCTTTACCAGGGTCTCAGTACA
GCCACCAAGGACACCTACGACGCCCTTCACATGCAGGCCCTGCCCCCTCGCGAAACGCGT

B10 Moderate-affinity:

GGATCCACCGCCATGGCCCTGCCCGTGACCGCCCTGCTGCTGCCCCTGGCCCTGCTGCTGCACGCCG
CCCGGCCTCAGGTGCAGCTGGTGCAGAGCGGCGGCGGCGTGGTGCAGCCCGGAGGCAGCCTGAG
GGTGAGCTGCGCCGCCAGCGGCGTGACCCTGAGCGACTACGGCATGCACTGGGTGCGGCAGGCTC
CCGGCAAGGGCCTGGAGTGGATGGCCTTCATCCGGAACGACGGCAGCGACAAGTACTACGCCGAC
AGCGTGAAGGGCCGGTTCACCATCAGCCGGGACAACAGCAAGAAGACCGTGAGCCTGCAGATGAG
CAGCCTGCGGGCTGAGGACACCGCCGTGTACTACTGCGCCAAGAACGGCGAGAGCGGCCCTCTGG
ACTACTGGTACTTCGACCTGTGGGGCAGGGGAACCCTGGTGACCGTGAGCAGCGGCGGCGGAGGC
AGCGGTGGCGGAGGCAGCGGCGGAGGCGGTAGCGAGATTGTGCTGACCCAGAGCCCTTCCTCT
CTGTCCGCCAGCGTGGGAGATAGAGTGACCATCACATGCCAAGCCTCCCAAGACATCTCCAA
CTACCTCAACTGGTACCAGCAGAAGCCCGGCAAGGCTCCCAAGCTGCTGATCTATGACGCCT
CCAATCTGGAGACCGGCGTCCCCTCCAGATTTTCCGGCAGCGGCTCCGGCACAGACTTTACC
TTCACCATCAGCTCTCTGCAGCCCGAGGACATCGCCACCTACTATTGCCAGCAGTATGATAA
TCTGCCTCTGACATTTGGCGGCGGCACCAAGGTGGACATTAAGGCGGCCGCCATCGAGGTGGA

GCAGAAGCTGATCAGCGAGGAGGACCTGCTGGACAACGAGAAGAGCAACGGCACCATCATCCACG
TGAAGGGCAAGCACCTGTGCCCCAGCCCCCTGTTCCCCGGCCCCAGCAAGCCCTTCTGGGTGCTGGT
GGTGGTGGGCGGCGTGCTGGCCTGCTACAGCCTGCTGGTGACCGTGGCCTTCATCATCTTCTGGGT
GCGGAGCAAGCGGAGCCGGCTGCTGCACAGCGACTACATGAACATGACCCCCCGGCGGCCTGGGC
CCACCCGCAAGCATTACCAGCCCTATGCCCCACCACGCGACTTCGCAGCCTATCGCTCCAGAGTGAA
GTTCAGCAGGAGCGCAGAcgCCCCCGCGTACCAGCAGGGCCAGAACCAGCTCTATAACGAGCTCAA
TCTAGGACGAAGAGAGGAGTACGATGTTTTGGACAAGAGACGTGGCCGGGACCCTGAGATGGGGG
GAAAGCCGAGAAGGAAGAACCCTCAGGAAGGCCTGTACAATGAACTGCAGAAAGATAAGATGGCG
GAGGCCTACAGTGAGATTGGGATGAAAGGCGAGCGCCGGAGGGGCAAGGGGCACGATGGCCTTT
ACCAGGGTCTCAGTACAGCCACCAAGGACACCTACGACGCCCTTCACATGCAGGCCCTGCCCCCTCG
CGAAACGCGT

2D4 Low-affinity:

GGATCCACCGCCATGGCCCTGCCCGTGACCGCCCTGCTGCTGCCCCTGGCCCTGCTGCTGCACGCCG
CCCGGCCTCAGGTGCAGCTGGTGCAGAGCGGCGGCGGCGTGGTGCAGCCCGGAGGCAGCCTGAG
GGTGAGCTGCGCCGCCAGCGGCGTGACCCTGAGCGACTACGGCATGCACTGGGTGCGGCAGGCTC
CCGGCAAGGGCCTGGAGTGGATGGCCTTCATCCGGAACGACGGCAGCGACAAGTACTACGCCGAC
AGCGTGAAGGGCCGGTTCACCATCAGCCGGGACAACAGCAAGAAGACCGTGAGCCTGCAGATGAG
CAGCCTGCGGGCTGAGGACACCGCCGTGTACTACTGCGCCAAGAACGGCGAGAGCGGCCCTCTGG
ACTACTGGTACTTCGACCTGTGGGGCAGGGGAACCCTGGTGACCGTGAGCAGCGGCGGCGGAGGC
AGCGGTGGCGGAGGCAGCGGCGGAGGCGGTAGCATCGTCCTCACACAGTCCCCTAGCACACTG
TCCGCTAGCGTGGGCGACAGAGTGACCATTACATGCAGAGCCTCCCAGAGCATTAGCAGCTG
GCTCGCTTGGTACCAGCAGAAACCCGGAAAAGCCCCCAAGCTGCTGATCTACAAGGCCTCCA
ACCTCCAATCCGGAGTCCCCTCCAGATTTTCCGGCTCCGGAAGCGGCACCGAATTCACACTG
ACAATCTCCTCTCTGCAGCCCGACGACTTCGCCTCCTACTACTGCCAGCAGTACTCCAATTA
CCCCCTCACCTTCGGCCAAGGCACCAAGGTGGAGATTAAGGCGGCCGCCATCGAGGTGGAGCA
GAAGCTGATCAGCGAGGAGGACCTGCTGGACAACGAGAAGAGCAACGGCACCATCATCCACGTGA
AGGGCAAGCACCTGTGCCCCAGCCCCCTGTTCCCCGGCCCCAGCAAGCCCTTCTGGGTGCTGGTGGT
GGTGGGCGGCGTGCTGGCCTGCTACAGCCTGCTGGTGACCGTGGCCTTCATCATCTTCTGGGTGCG
GAGCAAGCGGAGCCGGCTGCTGCACAGCGACTACATGAACATGACCCCCCGGCGGCCTGGGCCCAC
CCGCAAGCATTACCAGCCCTATGCCCCACCACGCGACTTCGCAGCCTATCGCTCCAGAGTGAAGTTC
AGCAGGAGCGCAGAcgCCCCCGCGTACCAGCAGGGCCAGAACCAGCTCTATAACGAGCTCAATCTA
GGACGAAGAGAGGAGTACGATGTTTTGGACAAGAGACGTGGCCGGGACCCTGAGATGGGGGGAA
AGCCGAGAAGGAAGAACCCTCAGGAAGGCCTGTACAATGAACTGCAGAAAGATAAGATGGCGGAG
GCCTACAGTGAGATTGGGATGAAAGGCGAGCGCCGGAGGGGCAAGGGGCACGATGGCCTTTACCA
GGGTCTCAGTACAGCCACCAAGGACACCTACGACGCCCTTCACATGCAGGCCCTGCCCCCTCGCGAA
ACGCGT

Supplementary Figure 1. Sequences of the CAR constructs used in this study, including high-affinity (B11), moderate-affinity (B10), and low-affinity (2D4) variants.

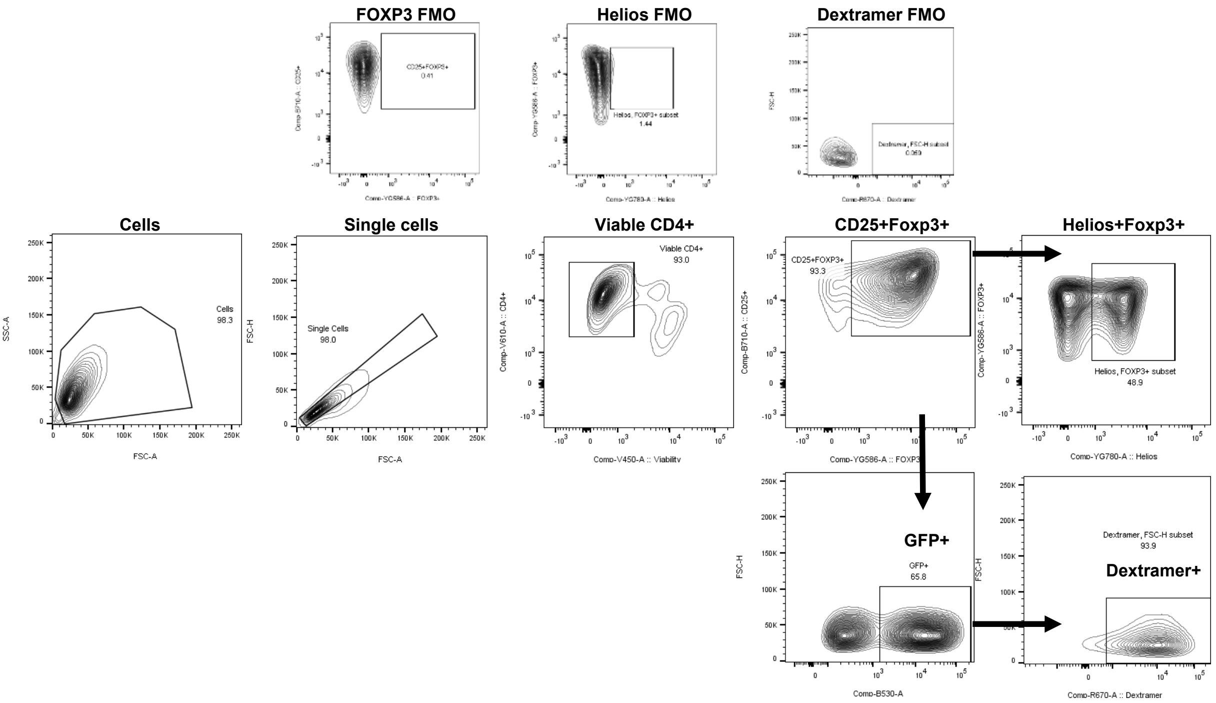

Supplementary Figure 2. Flow cytometry gating strategy used to assess CAR-Treg phenotype following expansion and stimulation. CAR-Tregs were sequentially gated on singlets, viable cells, $CD4^+$ lymphocytes and $CD25^+Foxp3^+$.

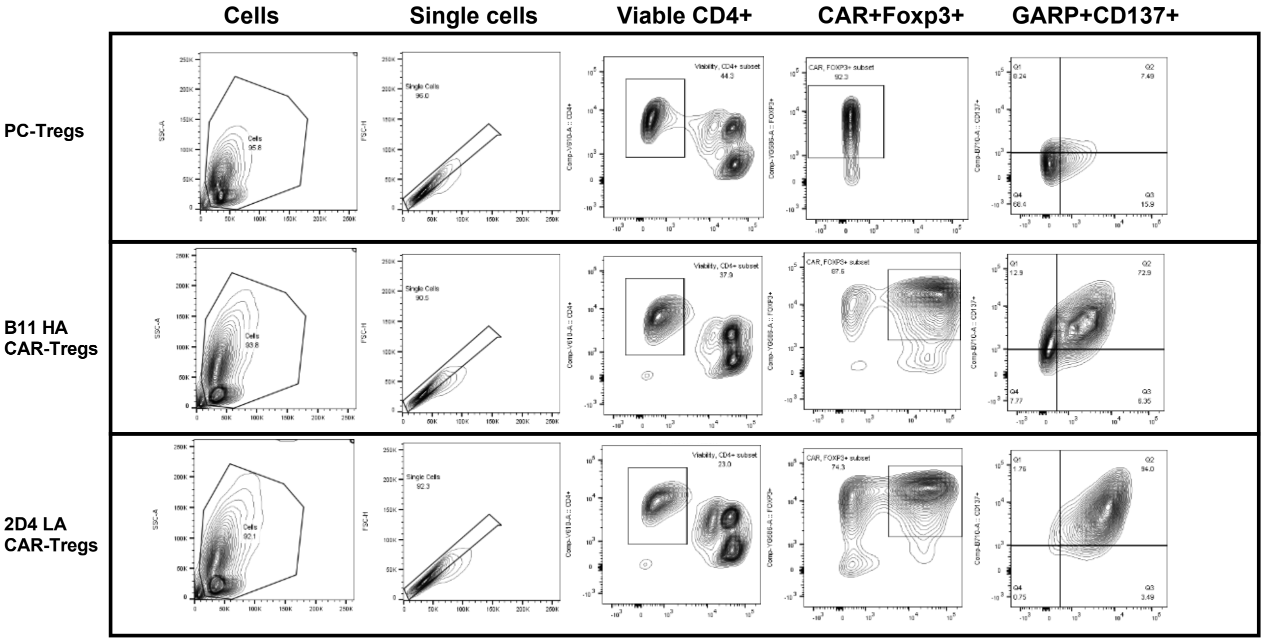

Supplementary Figure 3. Gating strategy used to identify antigen-specific CAR-Tregs in activation assays. Cells were gated on viable $CD4^+GFP^+$ CAR-Tregs and assessed for expression of activation markers following stimulation.

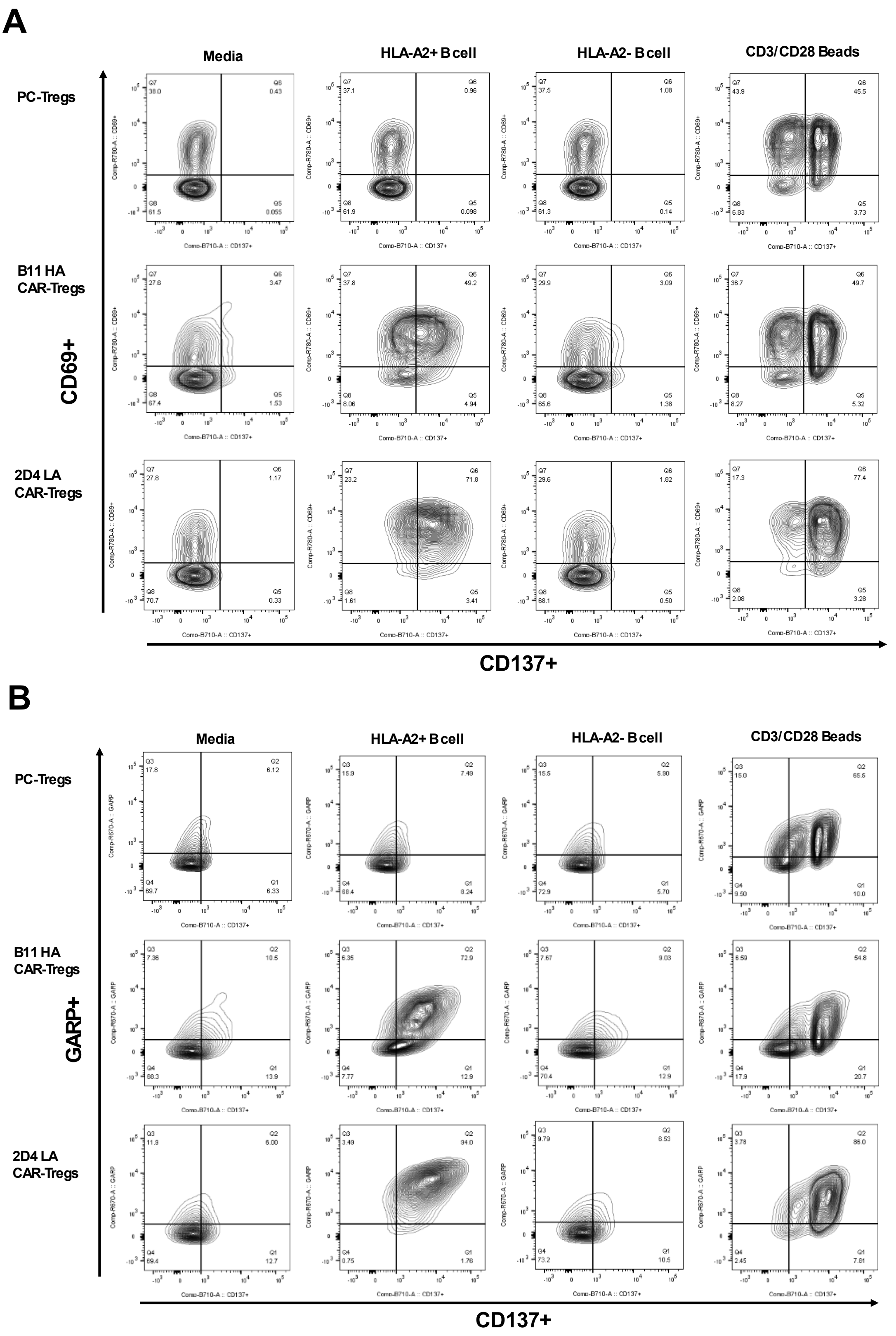

Supplementary Figure 4. Gating strategy used to quantify activated CAR-Tregs in control conditions. **(A)** Percentage of CAR$^+$ Tregs co-expressing CD69 and CD137. **(B)** Percentage of CAR$^+$ Tregs co-expressing CD137 and GARP.

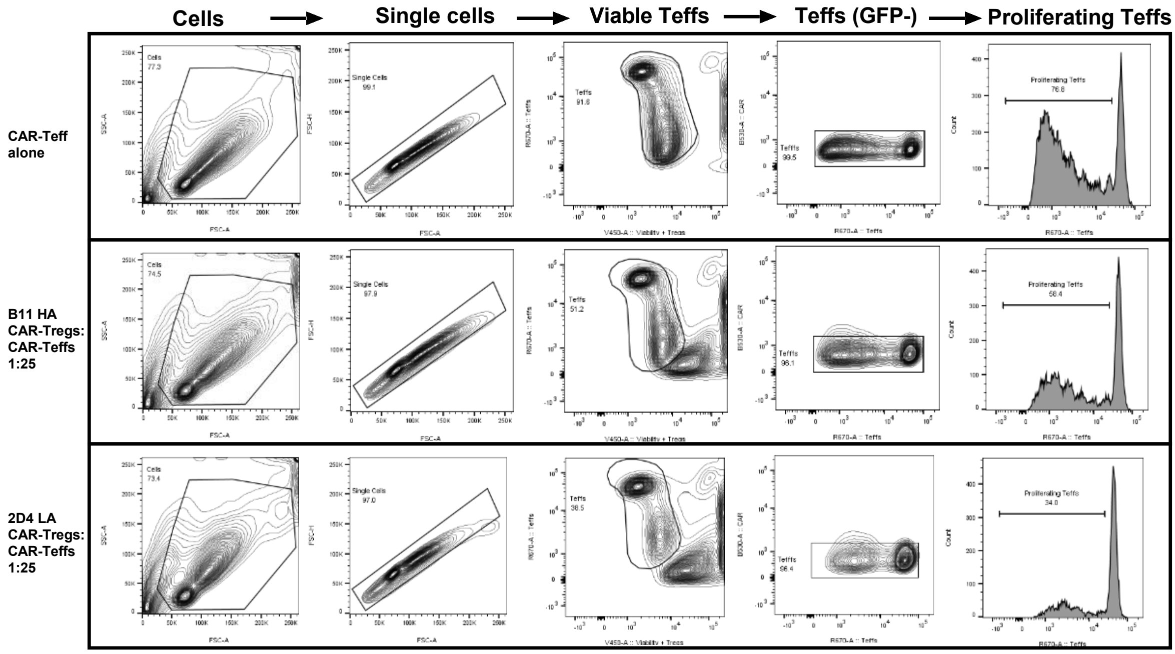

Supplementary Figure 5. Gating strategy used to quantify proliferating effector T cells in suppression assays. Proliferation was assessed by dilution of proliferation dye in Teff cells. Tregs and dead cells were excluded using a double-negative gating strategy. Representative plot shown for a 1:25 Treg:Teff ratio.

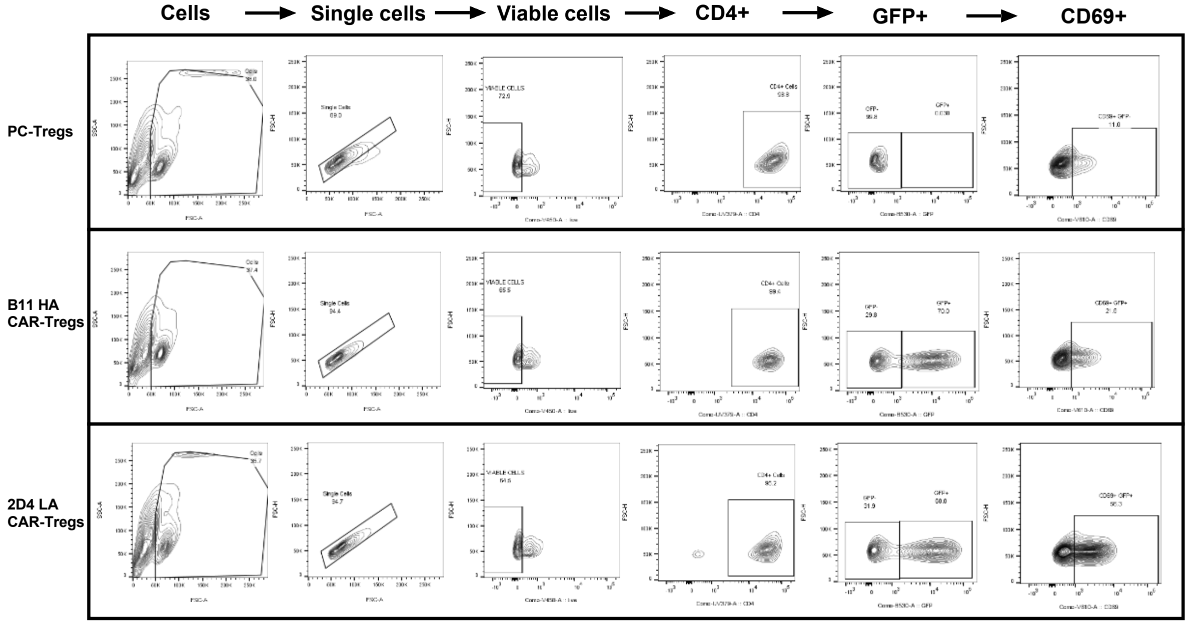

Supplementary Figure 6. Gating strategy used to identify activated CAR-Tregs following co-culture with HLA-A2$^+$ precision-cut liver slices. Cells were gated on viable CD4$^+$ GFP$^+$ CAR-Tregs prior to assessment of activation markers.

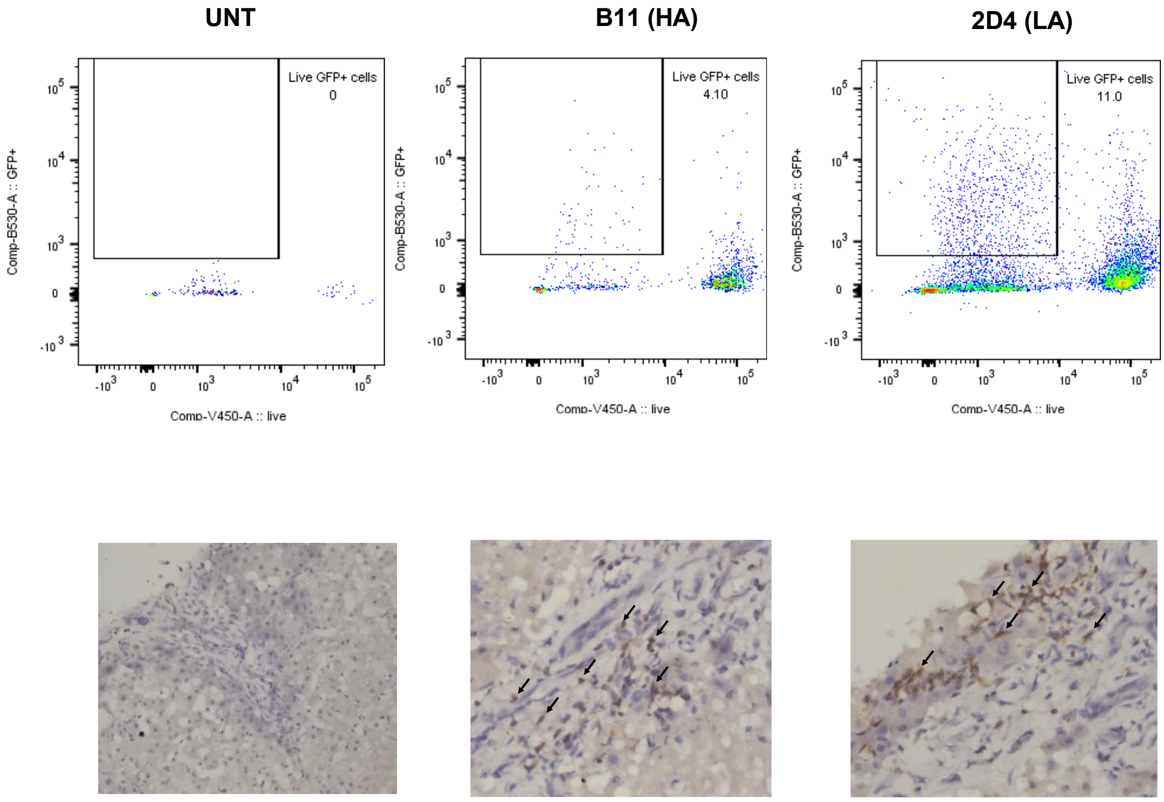

Supplementary Figure 7. Assessment of Treg infiltration into HLA-A2^{+} precision-cut liver slices. (A) Gating strategy identifying tissue-infiltrating CAR-Tregs. **(B)** Representative anti-GFP immunohistochemistry of tissue sections showing GFP^{+} CAR-Tregs (brown staining); arrows indicate positive cells within portal tracts.

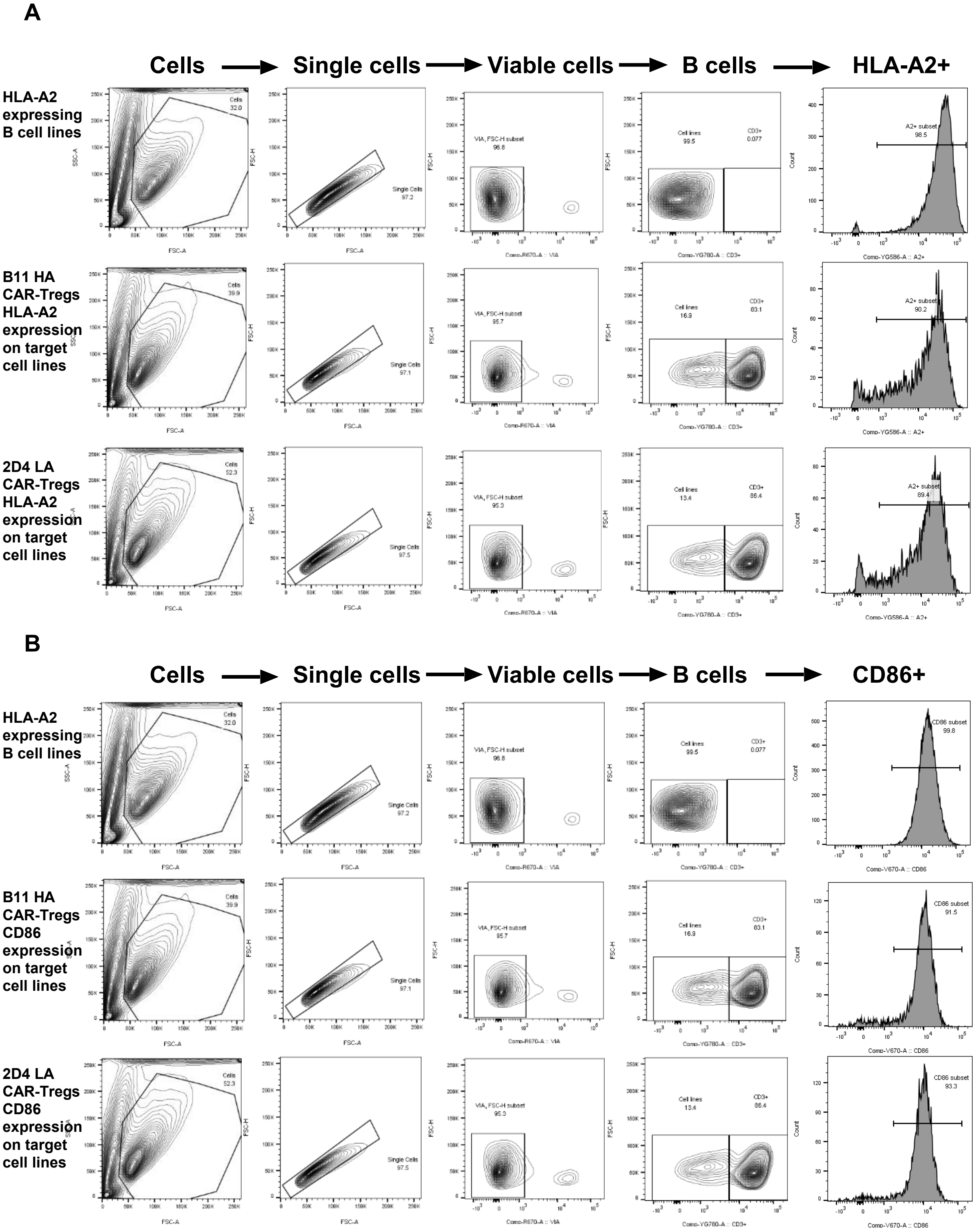

Supplementary Figure 8. Gating strategy used to assess trogocytosis in target B cell populations following co-culture. Gates are shown on target cells. **(A)** HLA-A2 expression. **(B)** CD86 expression following co-culture.

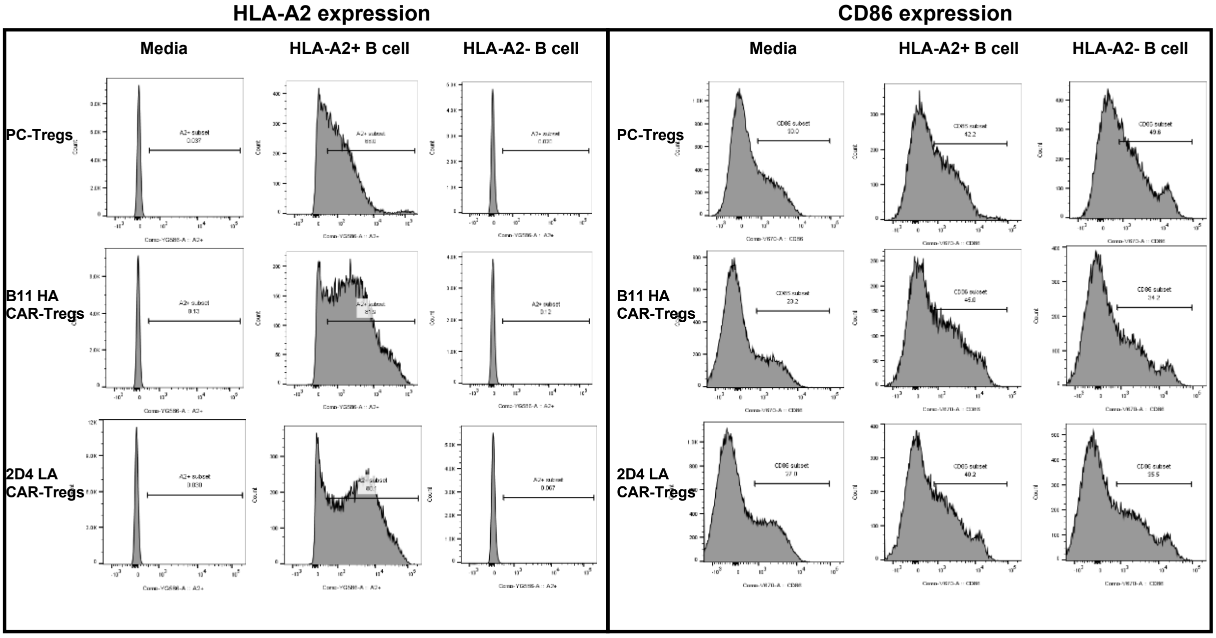

Supplementary Figure 9. Gating strategy used to assess acquisition of HLA-A2 and CD86 by Tregs following co-culture with target cells. Cells were gated on viable CD4$^+$ Tregs prior to analysis.

Supp Table 1: Antibodies used to assess the phenotype and stability of different Treg cell products.

TARGET	FLUOROCHROME	DETECTOR	VOLUME (µL)	SUPPLIER
Viability	V450	V450	1	Thermofisher
CD4	BUV395	UV379	2	BD Horizon
CD25	PerCPCy5.5	B710	5	BioLegend
GFP	--	B530	--	--
Dextramer (HLA-A*0201)	APC	R670	5	Immudex
FOXP3	PE	YG586	5	BioLegend
Helios	PE-Cy7	YG780	3	BioLegend

Supp Table 2: Antibodies used to assess the activation of alloreactive T cell clones.

TARGET	FLUOROCHROME	CATALOG NO	DETECTOR	VOLUME (µL)
VIABILITY	V450	TheroFisher, cat: 65086314	V450	1
CD4	V605	BD-Biosciences, cat: 562658	V610	2
CD69	APC-CY7	BD-Biosciences, cat: 557756	R780	2
CD127	V510	BD-Biosciences, cat: 563086	V525	3
CD137	Percp-Cy5.5	BioLegend, cat: 309813	B710	3
CAR/GFP	FITC	N/A	B530	N/A
GARP (7B11)	APC	Biolegend, cat: 35206	R670	5
GARP (G14D9)	APC	Invitrogen, cat: 50-9882-42	R670	5
FOXP3	PE	BioLegend, cat: 126404	YG586	5

Supp Table 3: Antibody panel for flow cytometric quantification of T-effs suppression.

TARGET	FLUOROCHROME	CATALOG NO	DETECTOR	VOLUME (µL)
VIABILITY + B cells (PROLIF)	V450	ThermoFisher, cat:65-0863-14	V450	1
CD4	BV605	BD-Biosciences, cat:562658	V610	3
CD25	PerCP 5.5	BD-Biosciences, cat:560503	B710	5
T-EFFs(PROLIF)	APC	ThermoFisher, cat:65-0840-85	R670	1
Tregs/CAR/GFP	FITC	N/A	B530	N/A

Supp Table 4: Antibody panel for identifying activation following PCLS co-culture.

TARGET	FLUOROCHROME	DETECTOR	VOLUME (µL)	SUPPLIER
VIABILITY	V450	V450	1	ThermoFisher,
CD4	BUV395	BUV395	3	BD-Biosciences,
CD69	APC-CY7	R780	2	BD-Biosciences,
CD137	Percp-Cy5.5	B710	3	BioLegend
CAR/GFP	FITC	B530	N/A	N/A
Dextramer	APC	R670	5	Immudex

Supp Table 5: Antibodies used to assess trogocytosis exhibited by CAR-Tregs.

TARGET	FLUOROCHROME	DETECTOR	VOLUME (µL)	SUPPLIER
Viability	APC	R670	1	Thermofisher
CD4	PE-CY7	YG780	2	BioLegend
Cell lines	V450	V450	1	eBioscience
CAR/Tregs	GFP	FITC	-	-
HLA-A2	PE	YG586	3	Biolegend
CD40	APC-CY7	R780	3	Biolegend
CD86	BV650	V660	3	BD Biosciences
HLA.DR	Percp Cy5.5	B710	2	Biolegend

Supplementary Methods

Histology and immunohistochemistry:

Haematoxylin and eosin (H&E) staining was performed on dewaxed paraffin-embedded tissue sections according to standard protocols (Kiernan, *Histological and Histochemical Methods*, 5th ed., Scion Publishing).

Immunohistochemistry was performed on sequential dewaxed paraffin-embedded sections. Endogenous peroxidase activity was quenched prior to heat-induced epitope retrieval. Sections were incubated overnight at 4 °C with a goat anti-GFP primary antibody (1:2000; Rockland, 600-101-215), followed by washing in Tris-buffered saline (TBS). A biotinylated secondary antibody was then applied (1:500; Vector Laboratories, BA-5000) for 1 hour at room temperature. After TBS washes, sections were incubated with streptavidin–HRP complex (Vector Laboratories, PK-6100) according to the manufacturer's instructions. Signal detection was achieved using an in-house DAB solution, yielding a brown precipitate at sites of GFP localisation. Sections were counterstained with Mayer's haematoxylin, dehydrated through graded alcohols, cleared in xylene, and mounted using DPX.
